# Modulation of fronto-parietal and autonomic activity underpins preserved working memory in middle-aged adults

**DOI:** 10.3389/fnagi.2026.1838829

**Published:** 2026-08-26

**Authors:** Myriam Cayre, Laure Spieser, Anaïs Guille, Nathalie Baril, Arnaud Weill, Caroline Chambon

**Affiliations:** Aix Marseille Univ, CNRS, CRPN, Psychology and Neuroscience Research Centre, Marseille, France

**Keywords:** attention, cognitive aging, event-related spectrum perturbation, inter-individual variability, locus coeruleus, pupil dilation, working memory

## Abstract

**Background:**

Cognitive aging is a heterogeneous process, often diverging from chronological age. However, it remains unclear when early signs of cognitive decline emerge and which neurophysiological markers distinguish age-related cognitive alteration from preserved performances. This study aimed to identify early indicators of cognitive versus chronological aging.

**Methods:**

In a group of middle-aged adults (MA, *n* = 60), we distinguished high performers (MA_HP; *n* = 24; mean age ± SEM 54.2 ± 0.6-year-old) from low performers (MA_LP; *n* = 24; mean age ± SEM 54.3 ± 0.7-year-old) considering their scores in a visuo-spatial working memory (VSWM) task. Both MA groups were compared to young adults (YA, *n* = 24; mean age ± SEM = 22.7 ± 0.5-year-old). Electroencephalography (EEG) and pupillometry were recorded while participants performed the WM task.

**Results:**

Our results showed that aging is accompanied by reduced pupil dilation peak. Yet, MA_HP exhibited prompt pupil dilation, comparable to YA, while MA_LP showed delayed and blunted pupil responses. Regarding EEG engagement index (EI), both MA groups exhibited significantly higher frontal EI than YA, while parietal EI was only increased in MA_HP compared to YA. Considering event related spectrum perturbation, aging was characterized by a loss of fronto-central and parietal theta event related depression (ERD) during the task. MA_HP distinguished themselves by parietal alpha and beta ERD during encoding and frontal theta, alpha and beta ERD during retention.

**Conclusion:**

Collectively, our data suggest that high WM performance in midlife depends on the flexible modulation of autonomic and cortical resources, potentially facilitating the attentional mobilization necessary for cognitive coping. These neurophysiological signatures allow clearer distinction between a chronological aging profile and a successful cognitive aging profile.

## Highlights

- Neurophysiological signature of cognitive aging is already detectable by midlife (50–60-year-old).
- Preserved working memory requires a youth-like functional LC-NE system
- Performance maintenance relies on increased engagement of the fronto-parietal network.
- Pre-frontal ERD within the 4–25 Hz range during retention is crucial for working memory maintenance.

## 1 Introduction

In the context of increasing life expectancy, understanding the mechanisms underlying cognitive aging has become a major societal and scientific challenge. While aging is typically associated with progressive decline in memory, executive function, reasoning, and processing speed, this process of cognitive aging is far from uniform. It is clearly established that, regarding brain functions, some individuals age better than others (LaPlume et al., 2022), suggesting that chronological age (the number of years lived) does not always match cognitive age which is the result of the cumulative impact of cellular processes on biological systems and their associated functional decline. In other words, an individual’s “chronological age” alone is not sufficient to predict his cognitive resources. Investigating both structural and functional brain characteristics is necessary to estimate an individual’s capacity to meet cognitive demands. For instance, neuroimaging studies have shown that some older adults retain brain volumes and white matter integrity comparable to those of much younger individuals (Gefen et al., 2015; Sun et al., 2016). However, structural properties and changes of gray- and white-matter account for a small (though significant) portion of the variability in cognitive trajectories during normal aging (Kalpouzos, 2025). Thus, functional properties, and notably neurophysiological metrics, may also contribute to explain different cognitive trajectories.

Among the cognitive domains impacted by age, working memory (WM) appears particularly sensitive (Naveh-Benjamin and Cowan, 2023). WM refers to the ability to temporarily store and manipulate information and is central to a broad range of higher-order cognitive functions (Baddeley, 2010; Barrouillet and Camos, 2015; Cowan, 2017, 2008). It is tightly linked to executive functions such as inhibition, cognitive flexibility, and updating (Guerrero et al., 2022; Hitch et al., 2025; Miyake and Friedman, 2012). The reduction in attentional resources associated with the decrease in information processing speed, typical of advancing age, would lead to a deterioration in the encoding and maintenance of information in WM, explaining a reduction in performance in older adults compared with young adults. This decline has been reported to begin as early as midlife (Brockmole and Logie, 2013). However, studies investigating cognitive abilities during midlife have yielded inconsistent findings. While some authors reported measurable declines beginning as early as the fourth or fifth decade of life (Salthouse, 2009; Singh-Manoux et al., 2012), others observed only subtle changes and emphasized the considerable heterogeneity and plasticity characterizing this period (Hughes et al., 2018; Lachman et al., 2015). We believe that one possible explanation for these discrepancies is that midlife represents a transitional period characterized by marked interindividual differences in aging trajectories, with individuals of similar chronological age exhibiting substantially different levels of cognitive preservation or decline (Ferreira et al., 2017; Park and Festini, 2016). Whether early neurophysiological changes associated with cognitive status can already be detected in middle-aged adults remains an open question.

Among neurophysiological markers, pupil dilation and EEG signature give interesting and complementary insight into brain functioning. Although pupil size can be affected by various conditions such as light intensity or unattended stimuli, pupillometry is now recognized as a reliable index of resource allocation in WM tasks (Rondeel et al., 2015). Task-evoked pupil responses have long been shown to correlate with WM load and individual performance (Kahneman and Beatty, 1966; Lenain et al., 2026; Unsworth and Robison, 2018), providing a non-invasive marker of neuromodulatory state and cognitive engagement and particularly mobilization of attentional resources. Unsworth and Robison (2018) theorized that the locus coeruleus (LC), a noradrenergic nucleus located in the brainstem involved in arousal and attention, plays a central role in cognitive processes related to WM. They proposed that LC dysregulation leads to larger tonic fluctuations, weaker phasic responses, and poorer activation of the fronto-parietal control network in low WM individuals. Therefore, pupil dilation emerged as a sensitive physiological index of attentional engagement. Phasic increases in LC norepinephrine (NE) in response to task demand indirectly triggers pupil dilation via the ventral medulla, therefore a growing number of studies have used pupil diameter as an indirect index of the activity of the LC-NE system (van der Wel and van Steenbergen, 2018). Despite its promise (Kim et al., 2024), pupillometry has rarely been combined with electroencephalography (EEG) in researches investigating the impact of aging on the WM and attention related fronto-parietal network activity.

Traditionally, EEG studies investigating the effect of aging have focused on oscillatory activity in specific frequency bands, such as theta, alpha, beta and gamma rhythms, which have been linked to attentional engagement. To assess cortical engagement in the WM task, we focused on two EEG metrics: event-related spectral perturbation (ERSP), and engagement index (EI). These metrics were selected because they reflect specific cognitive processes (e.g., attention engagement for EI, and active information processing for ERSP) and are sensitive to aging effects (Cummins and Finnigan, 2007).

Event-related spectral perturbation (ERSP) provides access to induced neural activity that is not phase-locked to stimulus onset and therefore may remain undetected using conventional event-related potential averaging, offering a more comprehensive characterization of cognitive processes such as attentional engagement. In fronto-parietal networks, oscillatory modulations in the theta (4–8 Hz), alpha (8–12 Hz), and beta (13–25 Hz) frequency bands have been consistently associated with WM load (Missonnier et al., 2011). Importantly, these oscillatory markers appear to be sensitive to the effects of aging (Deiber et al., 2010). ERSP thus constitutes a sensitive neurophysiological tool for characterizing age-related adaptations in the spatiotemporal dynamics of WM networks.

Since attention and workload are important components of WM performances, it is convenient to use electrophysiological proxies of these cognitive states (Berka et al., 2007; Kamzanova et al., 2014). The attentional engagement (EI), defined as the ratio of beta power on the sum of alpha and theta powers (Pβ/(Pα + Pθ)) at frontal and parietal locations, has been shown to be sensitive to attentional engagement into complex tasks (Pope et al., 1995). While pupil dilation is used as a proxy of the global autonomic arousal, EI offers temporal and spatial resolution to investigate the engagement of the fronto-parietal attention and WM related network. Many studies showed that the LC-NE influence on cortical activity is translated into modulation of alpha oscillatory activity which temporarily facilitate the flexible gating of information (Dahl et al., 2022, 2020; Marzo et al., 2014). However, the influence of age on this system is still unclear, some data showing a diminished alpha synchronization for irrelevant stimuli indicating a less efficient attentional filter (Deiber et al., 2010; Gazzaley et al., 2005) and other reporting equivalent alpha modulation in middle-aged and older adults (Tune et al., 2021). Again, these differing observations could be explained by the fact that only chronological age is considered in these studies.

A substantial body of evidence indicates that aging is associated with progressive alterations in attentional performance (Commodari and Guarnera, 2008). Neuroimaging studies indicate functional reorganization within the fronto-parietal attentional network, characterized by compensatory recruitment and altered connectivity patterns in middle-aged and older adults (Macpherson et al., 2014). Converging evidence further implicates age-related changes in LC-NE system, whose structural integrity and functional responsivity are critical for attentional modulation and arousal regulation (Dahl et al., 2023). Together, these findings suggest that age-related differences in attention reflect both large-scale network reconfiguration and altered neuromodulatory dynamics.

However, since midlife is related to an important interindividual variability (Lachman et al., 2015) resulting in heterogeneous cognitive patterns (Ferreira et al., 2017), we consider chronological age alone as a non-discriminating marker to study the effects of aging. In the present study, we thus aimed to investigate early changes in neurophysiological markers related to the attentional system in middle-aged adult. In this context, we hypothesized that “successful cognitive aging” (defined here as preserved WM performance at midlife, comparable to young adults) is characterized by a profile of activation of the fronto-parietal attentional system and a level of LC-NE activation that differs from that of age-related cognitive decline (defined here as altered WM performance compared to young adults).

## 2 Methods

### 2.1 Study participants

All participants have provided written, informed consent according to procedures approved by the local Ethics Committee, which followed the recommendations of the Declaration of Helsinki and received the authorization N° 21.01209.000020. This study included two age-group participants: 20–30-year-old (“young adults”, YA) and 50–60-year-old (“middle-aged”, MA). Young adults were recruited among Aix-Marseille university students whereas middle-aged were recruited by word of mouth and via announcement in a local journal. The participants received financial compensation of 40 euros. Apart from age, inclusion criteria were French level good enough to understand the instructions, normal vision (or corrected to normal). Exclusion criteria included neurological disease and ongoing treatments with molecules targeting the nervous system. All the participants were evaluated with the Mini-Mental State Examination (MMSE; Folstein et al., 1975) to ensure they have a non-pathological cognitive score (above 25 out of 30).

Besides, participants were asked not to drink exciting drinks (coffee, tea…) and not to smoke before coming to the laboratory to perform the cognitive exercises.

**FIGURE 1:**
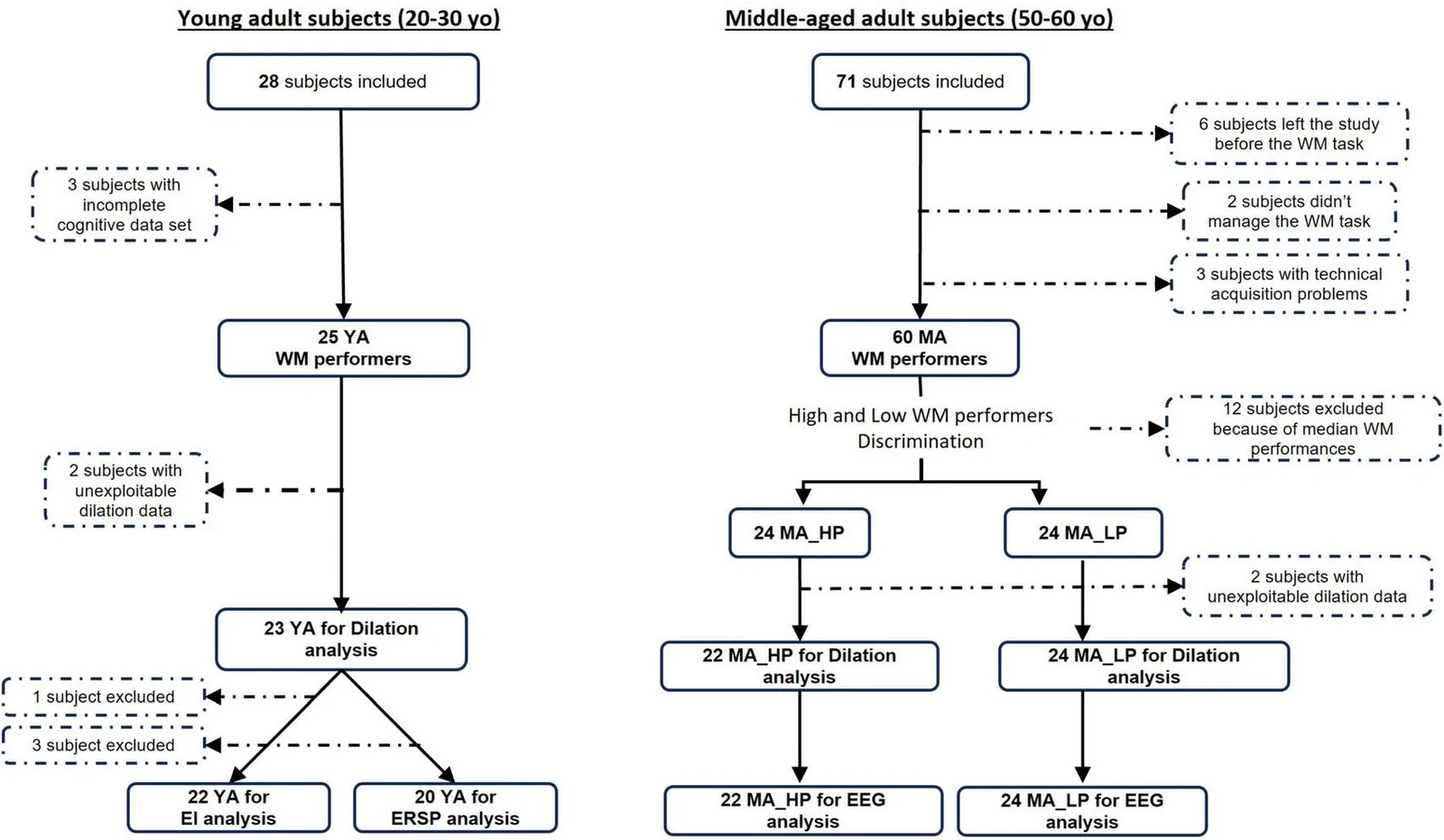
Flow chart of the experiment. Number of participants included and considered for analyses at each step of the study. EEG, electroencephalography; YA, young adults; MA, middle-aged adults; HP, high performers; LP, low performers; WM, working memory.

Overall, we recruited 28 young adults (mean age ± SEM: 22.9 ± 0.5-year-old; ratio F/M: 17/11) and 71 middle-aged participants (mean age ± SEM: 54.2 ± 0.4-year-old; ratio F/M: 45/23). Depending on the measure considered the number of subjects included in the analysis varied. A flow chart is provided to describe the number of subjects included at each step (Figure 1). For the correlation analysis, only subjects with a complete set of data were considered.

### 2.2 Visuo-spatial working memory task (VSWM)

The task was computerized and presented on an LCD Gamer screen AOC 23.8” with 120Hz refreshing speed, a 10% contrast, a 5% brightness and a grey background screen (rgb 0.3; 0.3; 0.3). These adjustments were chosen to follow the recommendation from Tsukara and Engle (2021) to allow the best variability of the pupil size. Answers were given using a Cedrus^®^ keyboard to improve RT accuracy.

The task was developed on Psychopy3 software. It started with the written instructions, then a training session of 20 trials was proposed to the participant, allowing him to get familiar with the task and allowing the experimenter to check that the instructions were correctly understood. In case of difficulties, the subject had the possibility to do the training again. Finally, the real task started.

**FIGURE 2:**
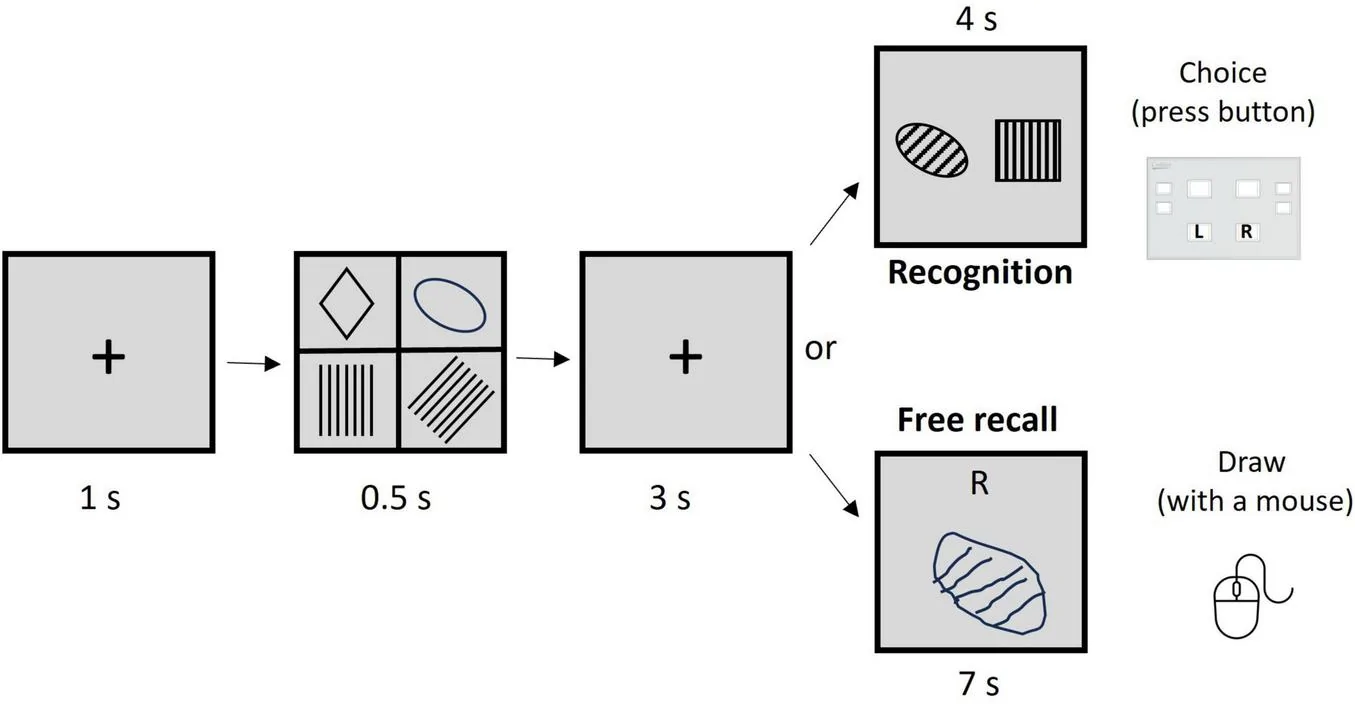
Schematic description of the visuo-spatial working memory task. The task consists in a series of 4 screens. (1) A fixation cross is presented (1 s) to focalize the attention of the subject at the center of the screen; (2) 4 stimuli are presented (0.5 s). Two shapes (out of eight possible) are presented in the top row and two stripe patterns (out of four: horizontal, vertical, diagonal right and diagonal left) in the bottom row; (3) a fixation cross is presented during the “retention” period (3 s), during which participants have to mentally construct a figure from the two elements on the right and another figure from the two elements on the left (shape + stripes); (4) in the recognition version of the task, two possible answers are proposed and the participant has to click on the correct one (there is always one); in the free recall version, a message ‘draw L’ or “draw R” appears on the top of the screen, indicating whether to draw the figure created mentally from the shape and the stripes on the right (R) or left (L). The participant has a maximum of 7 s to draw the answer with the mouse.

Each trial of the task consisted of a succession of 4 screens (Figure 2): 1- A black fixation cross (8 mm size) presented for 1s to get participants into focus. 2- A big square divided into four squares of 42 mm each side, showing two shapes (out of eight possible: square, circle, triangle, diamond, trefoil, infinity symbol, ellipse, clepsydra) in the top row and two stripe patterns (out of four: vertical, horizontal, diagonal-up, diagonal-down) in the bottom row; stimuli were shown for 500 ms. 3- A fixation cross presented for 3 s during the “retention” period, when participants had to mentally construct a figure from the two elements on the right (right shape + right stripes) and another figure from the two elements on the left (left shape + left stripes). 4- The response screen, which duration depended on the version of the task, i.e., 4 s for the recognition version and 7 s for the free recall version. If the participant did not respond within the delay, a non-response was counted and the task moved on to the next trial.

In the recognition version, the participant had to choose the correct answer from two proposals, by pressing the “right” or “left” key on the Cedrus^®^ keyboard. In the free recall version, the participant had to draw the correct answer on the screen using the computer mouse. In this case, a message ‘draw L’ or “draw R” appeared on the top of the screen, indicating the participant whether to draw the figure created mentally from the shape and the stripes on the right (R) or left (L).

The task was divided into 8 blocks of 24 trials each, alternating ‘keyboard’ blocks (recognition version) and ‘drawing’ blocks (free recall version). Thus, for each version, a total of 96 trials were proposed. Inter-trial-intervals (between 2.5 and 3 s) were inserted in order to prevent the subject from predicting the occurrence of the next trial as well as to allow the pupil to return to its baseline before starting the next trial. Performance was assessed by the percentage of correct responses and response time (RT) for the recognition version and only by the percentage of correct responses for the free recall version. A response was deemed correct only if both the shape and the stripe orientation matched the presented stimuli. Graphical precision (e.g., line regularity, proportions) was not considered in the scoring, as mouse-based drawing inherently limits accuracy. However, since the proposed shapes and stripe orientations were sufficiently distinct, the identification of participant responses remained unambiguous. Even in cases of approximate drawing, the response given was always clear, and scoring was not exposed to subjectivity, thus double-rater scoring was not necessary.

### 2.3 Data collection and analysis

Pupil dilation and EEG were recorded throughout the VSWM task.

#### 2.3.1 Pupil dilation recording and analysis

Participants’ pupillary dilation was measured throughout the task, using a binocular eye tracker (Live Track Lightning – Cambridge Research System^®^) with a sampling frequency of 125 Hz. The participant-screen distance (D = 88 cm) and participant-eye tracker distance (d = 30 cm) were defined according to the manufacturer’s recommendations. Luminosity of the room in which the experiment took place was measured at 18 lux at screen level, allowing adequate pupil dilation variations. The head position was stabilized with a chin rest and forehead support throughout the task. Pupil diameter was calibrated and expressed in mm. We report eye-tracking results for the left eye, since both eyes were highly reliably correlated (*r* = 0.989; *p* < 0.0001). The extracted values correspond to the horizontal axis of the pupil, which is least affected by artifacts compared with the vertical axis. The periods of signal loss corresponding either to blinks or a system acquisition fault were removed from the analysis. Invalid data (values < Q1–1.5D or values > Q3+1.5D, where Q1 and Q3 are the first and third quartile respectively, and D = Q3–Q1) were also removed. Trials with more than 20% missing data were then excluded from the analysis.

Pupil dilation was analyzed using baseline subtraction (i.e., absolute change from baseline) rather than percentage change to preserve interindividual variability in pupillary responses and avoid mathematical artifacts associated with ratio-based normalization, particularly when baseline values differ between groups (Mathôt et al., 2018; Strauch et al., 2022). This approach ensures that differences in cognitive effort between high performers (HP) and low performers (LP) are accurately reflected. For each trial, pupil diameter was averaged over the last 500 ms of the pre-stimulus fixation cross to establish a baseline. This baseline was subtracted from pupil diameter values recorded during the stimulus presentation and retention periods, yielding task-evoked pupillary responses for each trial. The dynamics of pupillary dilation following the stimuli presentation was analyzed, using 200 ms bins, between the stimuli presentation and the end of the retention delay, thus during a period of 3.5 s. The maximum value of pupillary dilation for each trial was extracted and then averaged over all trials per participant. Similarly, the latency to reach this maximum was extracted for each trial and averaged over all trials for each participant. To ensure a robust and physiologically plausible estimation of pupil dilation onset at the single-trial level, onset was defined as the first time point at which pupil size exceeded two standard deviations of the trial-specific baseline for at least two consecutive time bins (i.e., ≥400 ms). This criterion minimizes spurious detections due to baseline noise and enforces temporal persistence consistent with the known latency of cognitively driven pupillary responses (Mathot et al., 2014; Rajkowski et al., 2004). The onset latency for each participant was defined as the average onset across all trials.

#### 2.3.2 EEG recording and pretreatment

In order to limit the impact of surrounding electrical equipment on the signal quality, the participants were placed in a Faraday cage for the duration of the experiment. Brain activity was recorded during the tasks using a Biosemi Active Two system (Biosemi Instruments^®^, Amsterdam, Netherlands) with 64 electrodes, international 10-20 system and a sampling frequency of 2048 Hz. Nasion-inion and pre-auricular anatomical distances were measured to locate and mark the vertex of each individual. Four external electrodes were used to detect eye movements. The impedance of the electrodes was kept below 20 kOhms.

Filtering, segmentation and data cleaning inspection and analysis were performed using the EEGLab Toolbox for MATLAB using MATLAB 2024b (The MathWorks Corp) (Delorme and Makeig, 2004). A resampling at 512 Hz and two bandpass filters were applied: a 0.5 Hz high-pass filter a 40 Hz low-pass filter. The noisy signals outside periods of interest were manually rejected. The data was then referenced to the mean reference using the PREP pipeline. This pipeline is an EEGLAB plugin that allows the calculation of a robust reference in an iterative fashion, by subtracting the average of all the valid electrodes from each electrode (Bigdely-Shamlo et al., 2015). Electrodes with a poor signal were detected and interpolated using EEGLAB’s spherical spline function. An independent component analysis was then performed using the RUNICA algorithm. The components clearly identified as eye movements were removed. Trials containing artifacts were visually identified and rejected.

#### 2.3.3 EEG analysis

Analyses focused on three time-intervals: fixation (“fix”; 1s long), stimulus presentation (“stim”; 0.5 s long), and retention (“ret”; 3 s long), which was also subdivided into two windows (ret1: 0–1.2 s, ret2: 1.2–3 s) based on pupil dynamics.

Continuous EEG data were epoched from -1 to 3.5 s relative to stimulus onset. Spectral power was first estimated in EEGLAB using the *spectopo* function, and power values were then averaged within the theta (4–8 Hz), alpha (8–12 Hz), and beta (13–22 Hz) bands. These band-specific estimates were subsequently used to compute the Engagement Index (EI = Pβ/(Pα + Pθ)) either in the frontal (Fz) or parietal area (sum P1, Pz, P2). EI has been extensively validated as electrophysiological markers of attentional engagement. EI’s sensitivity to task difficulty has been confirmed in both laboratory and applied settings (Berka et al., 2007; Kamzanova et al., 2014; Pope et al., 1995). EI complements pupil dilation as an index of task engagement by providing fine-grained temporal resolution and by capturing activity within fronto-parietal networks, which are critical for VSWM.

In parallel, time–frequency decomposition was performed in EEGLAB using the *newtimef* function with Morlet wavelets (wavelet parameters: [3 0.5]) over a frequency range of 3–30 Hz. ERSPs were computed at the prefrontal (FPz), frontocentral (FCz), and parietal (Pz) electrodes. We selected *a priori* these three midline electrodes based on our hypotheses and on the well-established frontoparietal organization of working memory and attention networks, rather than through an exploratory whole-scalp approach. Frontal midline sites (FPz and FCz) are particularly sensitive to executive control processes and frontal-midline theta activity originating from medial prefrontal and anterior cingulate regions (Hsieh and Ranganath, 2014), whereas the parietal midline electrode (Pz) captures posterior alpha activity associated with visuospatial storage, attentional maintenance, and working memory processing (Bertaccini et al., 2022). Thus, this choice was hypothesis-driven rather than data-driven and allowed us to test our specific predictions while limiting the number of statistical comparisons. Numerous studies have shown that frontal theta and posterior alpha activity recorded over these midline regions constitute robust electrophysiological markers of cognitive load and visuospatial working memory performance (Nakamura-Palacios et al., 2023; Zhozhikashvili et al., 2022).

ERSP was baseline-corrected log-power changes relative to the -1 to 0 s pre-stimulus interval. This approach yields trial-averaged spectral power changes. For each electrode, condition and participant, ERSP values were extracted in the previously defined frequency bands.

### 2.4 Statistical analyses

Data analyses were conducted using Prism (GraphPad Software) and JASP. Group comparisons were carried out using one-way ANOVA, two-way or three-way repeated measures ANOVA. When normality was not respected, Kruskal Wallis tests were used instead of ANOVA. When the assumption of sphericity was violated, the Greenhouse-Geisser correction was applied. For datasets containing missing values, repeated-measures analyses were performed using a mixed-effects model fitted by restricted maximum likelihood (REML). In accordance with the implementation recommended in GraphPad Prism, Greenhouse–Geisser-adjusted degrees of freedom were used when the assumption of sphericity was not met. In *post-hoc* comparisons, Holm and Tukey corrections were applied. For correlation analyses, Pearson’s or Spearman’s correlation coefficients were calculated, depending on whether the data followed a Gaussian distribution. A *p*-value less than 0.05 was considered as statistically significant. When we performed two-way ANOVAs after a three-way ANOVA, i.e., for the ERSP analysis, we applied a Bonferroni correction to the α value such as the significance threshold was considered at 0.016 (α = 0.05/3) instead of 0.05.

One-sample *t*-tests were applied to estimate whether dilation values data were significantly different from baseline; *p* values were corrected with FDR correction for 52 comparisons. One-sample *t*-tests were also applied to estimate whether ERSP data were significantly different from baseline (event related synchronization (ERS) or event related synchronization (ERD)); *p* values were corrected with FDR correction for 27 comparisons.

## 3 Results

### 3.1 WM performance distinguishes high and low performers in middle-aged adults

**FIGURE 3:**
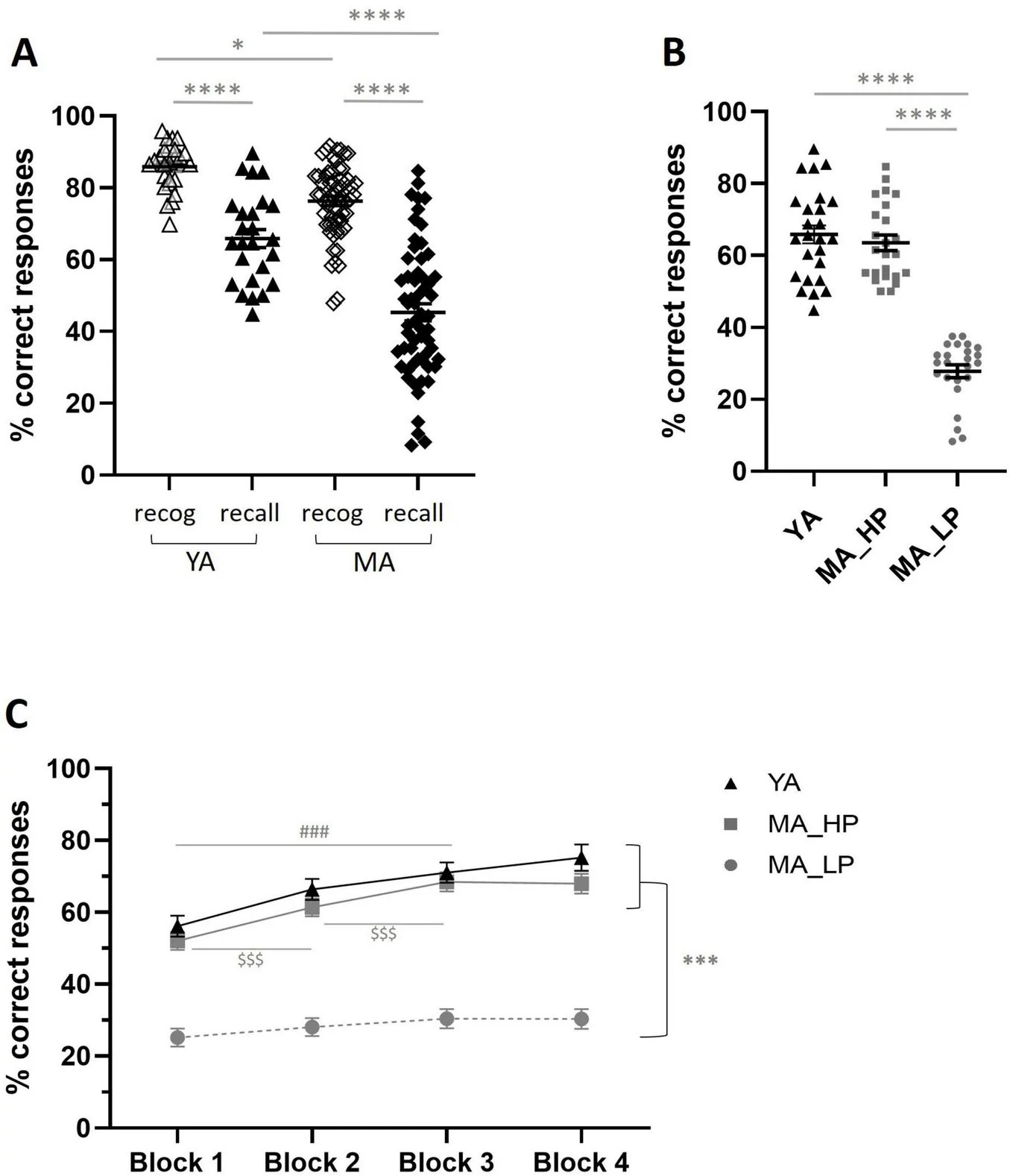
Performances in the working memory task. **(A)** Percentage of correct responses in the visuo-spatial working memory (VSWM) task for the young adult group (YA) and the middle-aged adult group (MA) in the two versions of the task, the recognition version (“recog”) and the free recall version (“recall”). Each dot represents an individual participant score; mean ± SEM is also shown for each group. **(B)** Percentage of correct responses in the free recall version of the VSWM task for the YA and for the two subgroups of MA participants: high and low performers (MA_HP and MA_LP respectively) discriminated based on the median score of the group ± confidence interval (90% CI). Each dot represents an individual participant score; mean ± SEM is also shown for each group. **(C)** Evolution of the percentage of correct responses across the VSWM task blocks. YA and MA_HP improve their scores along the task whereas MA_LP do not. YA, young adults (*n* = 24); MA_HP, middle-aged adult high performers (*n* = 24); MA_LP, middle-aged adult low performers (*n* = 24). Data are expressed as mean ± SEM. *p < 0.05; ***p < 0.001; ****p < 0.0001 for between group comparison. ^###^*p* < 0.001 for between blocks comparison within YA group. ^$$$^*p* < 0.001 for between blocks comparison within MA_HP group.

Among the 28 young adults (YA) and 71 middle-aged adults (MA) included in this study, 25 and 60 respectively performed the VSWM task (Figure 1). The task was proposed in two different versions: a recognition version in which the participant had to choose the good response among two propositions (key press) and a free recall version in which he/she had to produce the response (drawing). As expected, the two-way repeated-measures ANOVA revealed a significant main effect of task version, indicating that performance was lower in the free recall compared to the recognition version across both age groups [F (1,83) = 241.1, *p* < 0.0001]. A significant main effect of age group was also observed, with YA outperforming MA overall [F (1, 83) = 29.4, *p* < 0.0001] (Figure 3A). Importantly, the group × task version interaction was significant [F (1, 83) = 11.6, *p* = 0.0013], indicating that the performance gap between YA and MA was more pronounced in the free recall condition (predicted mean difference = 20.6) than in the recognition condition (predicted mean difference = 9.6). These results indicate a stronger age-related performance decline in more demanding condition, as soon as middle-age.

We therefore chose to focus the remainder of the study on the free recall version of the task, in order to explore potential neurophysiological underpinnings. Notably, we observed that interindividual variability in free recall performance was twice as high in MA compared to YA, with coefficients of variation of 40.5% and 19.1%, respectively. This suggests a heterogeneous performance profile within the MA group. To identify physiological markers that may allow to distinguish high from low performers, we divided MA participants into two subgroups based on the median free recall performance score (median = 43.5% correct responses), using a 90% confidence interval (37.5%; 50.0%). We thus excluded 12 participants whose scores were between 37.5 and 50% good responses (Figure 3B). Interestingly, middle-aged high performers (MA_HP; N = 24) exhibited WM performances similar to YA (63.5 ± 2.2% vs. 65.4 ± 2.6% correct responses respectively, *p* = 0.8181), whereas middle-aged low performers (MA-LP; N = 24) obviously showed performances significantly lower than both YA and MA-HP groups (27.9 ± 1.8%; *p* < 0.0001).

Table 1 shows the mean demographic characteristics of YA, MA_HP and MA_LP participants. An ANOVA performed on the MMSE data revealed a main effect of group (F = 4.123, *p* = 0.021). *Post hoc* comparisons indicated that MA_LP participants had significantly lower MMSE scores than MA_HP participants, although all individual scores in both middle-aged groups remained above 28. Scores above 28 indicate no cognitive impairment. The slight difference between YA and MA_LP is not clinically meaningful but reflects normal variability in this test.

**TABLE 1:** Demographic characteristics of the three populations.

Characteristics	YA (*n* = 25)	MA_HP (*n* = 24)	MA_LP (*n* = 24)
Age (years)	22.9 ± 0.5	54.2 ± 0.6	54.3 ± 0.7
Female/Male Ratio	15/9	14/10	15/9
School years	16.0 ± 0.3	16.4 ± 0.7	15.4 ± 0.6
MMSE score (/30)	28.9 ± 0.3	29.2 ± 0.2	28.2 ± 0.4*

Summary of the demographic characteristics of the three populations tested. *p < 0.05 for the comparison MA_LP vs. MA_HP. Data are expressed as mean ± SEM. YA, Young adults; MA_HP, Middle aged high-performer; MA_LP, Middle-aged low-performer. MMSE, Mini Mental State Examination.

We then examined whether the evolution of WM performance over time (across blocks) differed between the three groups (Figure 3C). A mixed-effects model (REML) revealed a significant main effect of time, indicating an overall improvement in performance across the four blocks [F (1.887, 45.28) = 88.52, *p* < 0.0001; Greenhouse-Geisser corrected]. A significant main effect of group was also observed [F (2.002, 48.06) = 23.91, *p* < 0.0001; Greenhouse-Geisser corrected]. Turkey’s multiple comparison tests showed that in all four blocks, YA and MA_HP showed similar performances while MA_LP exhibited reduced success compared to both groups (*p* < 0.0001 in both cases). The group × time interaction was significant [F (4.278, 94.82) = 2.367, *p* = 0.05; Greenhouse-Geisser corrected], suggesting that the trajectory of improvement differed across groups. Indeed, the MA_LP group did not significantly improve over the four blocks (*p* > 0.2822), whereas the MA_HP group showed significant gains between each of the first three blocks (block 1 vs. 2 *p* = 0.004, and block 2 vs. 3, *p* = 0.021), followed by a plateau between blocks 3 and 4 (*p* = 0.9889). The YA group also exhibited a significant improvement between block 1 and block 3 (*p* = 0.0006).

In summary, while both YA and MA_HP participants demonstrated learning-related gains in WM performance over time, the MA_LP group showed reduced performance from the start and no evidence of improvement.

We next evaluated the cognitive engagement during the WM task using physiological recordings: the task-evoked pupillary responses and an EEG index of engagement index (EI).

### 3.2 Pupil dilation dynamics distinguishes between high and low middle-aged performers

**FIGURE 4:**
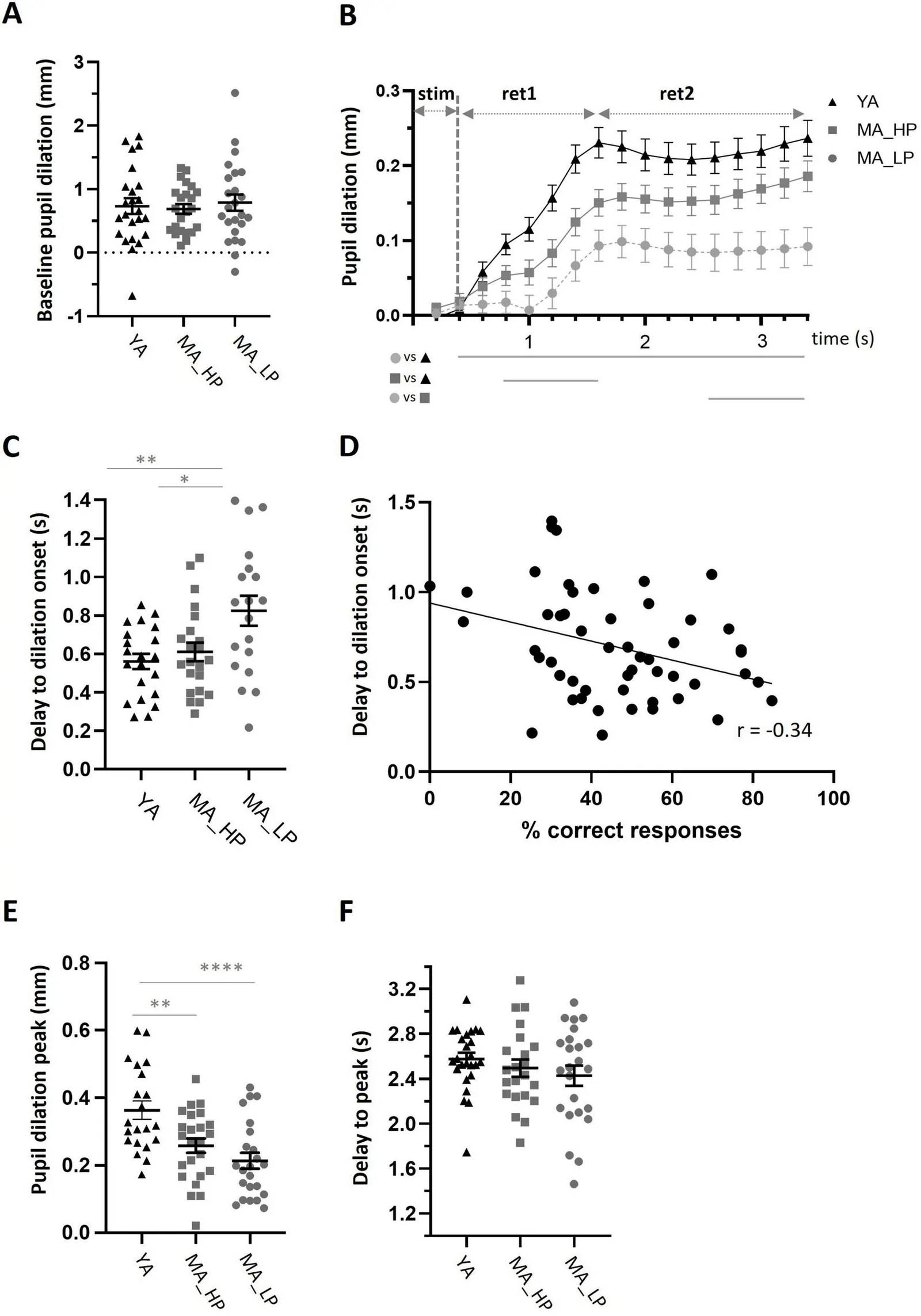
Pupil dilation during the visuo-spatial working memory task. **(A)** Baseline dilation quantified during the fixation cross. Baseline dilation was calculated by subtracting the dilation value quantified during a resting period to the dilation quantified during the fixation cross at the beginning of the trial. No difference was observed between the three groups. **(B)** Dynamic of the pupil dilation across a trial. The curves represent the variation of the pupil diameter after stimuli presentation, using as baseline the diameter measured during the fixation cross. stim = stimulus presentation; ret1 = first period of retention characterized by fast dilation; ret2 = second period of retention characterized by a plateau. Horizontal lines below the graph indicate periods of significant differences between groups. MA_LP showed a significantly lower dilation than YA throughout retention while MA_HP differed from YA only during ret1. MA_LP showed reduced dilation compared to MA_HP only at the end of the retention period. **(C)** Delay to dilation onset. Dilation latency is increased in MA_LP compared to both YA and MA. **(D)** Correlation between the delay to dilation onset and working memory performances in elderly. A significant negative correlation indicates that the latter the pupil starts dilation, the worse is the working memory score. **(E)** Pupil dilation peak in the three groups. Both MA_HP and MA_LP showed a lower dilation peak than YA, indicating an impact of age. **(F)** Delay to the pupil dilation peak. No difference was observed between the three groups. YA = young adults (*n* = 24); MA_H*P* = middle-aged adult high performers (*n* = 22); MA_L*P* = middle-aged adult low performers (*n* = 24). Data are expressed as mean ± SEM. *p < 0.05; **p < 0.01; ****p < 0.0001.

We recorded pupil diameter both during a resting-state period and while participants performed the WM task. Compared to resting state, the preparatory period before stimulus presentation (i.e., during the first fixation cross, when no cognitive processing was required) elicited pupil dilation in all three groups (Figure 4A), with no difference between groups [F (2, 68) = 0.1926, *p* = 0.8253], indicating a similar level of task engagement across groups.

We used four task-evoked pupillary responses: (1) dynamic dilation following stimulus presentation and during retention; (2) delay to dilation onset; (3) peak dilation (i.e., maximum dilation within each trial); and (4) peak latency, which is the time elapsed between probe onset and maximum dilation. Pupil dilation following stimulus presentation and during the retention phase revealed a biphasic pattern: an initial rapid increase during the first 1.2 s of retention (ret1), followed by a plateau from 1.2 to 3 s (ret2). A two-way repeated-measures ANOVA showed a significant main effect of time [F (2.413, 159.3) = 90.33, *p* < 0.0001; Greenhouse-Geisser corrected], a significant main effect of group [F (2, 66) = 9.844, *p* = 0.0002], and a significant time × group interaction [F (32, 1056) = 5.372, *p* < 0.0001], indicating group differences in the temporal dynamics of pupil dilation (Figure 4B). *Post-hoc*-tests revealed that MA_LP exhibited significantly lower pupil dilation compared to YA throughout the entire retention period (all fifteen *p*-values ranging between 0.0001 and 0.005). In contrast, MA_HP only showed reduced dilation relative to YA during a brief window between 800 ms and 1.2 s after stimulus offset (*p*-values between 0.018 and 0.039). Notably, during the final 600 ms of the retention phase, MA_HP was the only group to keep dilating (*p* < 0.001) and thus reached values significantly greater than those of the MA_LP group (*p*-values between 0.004 and 0.021).

We further determined the timepoint when dilation became significantly different from baseline after stimulus presentation (dilation latency). It differed between groups [F (2, 66) = 7.904, *p* = 0.003; Figure 4C], with greater dilation latency observed in MA_LP (0.824 ± 0.062 s) compared to both YA (0.561 ± 0.036 s; *p* = 0.0011) and MA_HP (0.611 ± 0.048 s; *p* = 0.0106). No significant difference was found between MA_HP and YA (*p* = 0.7688). Furthermore, we observed a significant negative correlation between dilation latency and VSWM performance among middle-aged participants (r = −0.34, *p* = 0.0125; Figure 4D). These results suggest a performance-related rather than an age-related effect on dilation latency during the WM task.

Independently of time course and dynamics, maximum dilation (or dilation peak) also differed between the groups [F (2,66) = 10.62, *p* = 0.0001; Figure 4E], with greater dilation observed in YA compared to both MA_HP (*p* = 0.0151) and MA_LP (*p* < 0.0001). No significant difference was found between MA_HP and MA_LP (*p* = 0.2498), suggesting an age-related, rather than a performance-related, effect on maximum pupil dilation during the WM task.

By contrast, the time to reach peak dilation did not differ significantly between groups (*p* = 0.3734; Figure 4F), suggesting that despite the initial dilation delay in MA_LP, the peak occurred within similar time frame.

Together, these findings demonstrate distinct pupil dilation dynamics across the three groups during the retention phase of the WM task. While MA_HP followed the temporal profile of dilation observed in YA (although with a smaller amplitude), MA_LP showed both a delayed dilation onset and a substantially reduced level of dilation, indicative of reduced or less efficient cognitive engagement.

### 3.3 Enhanced parietal engagement characterizes middle-aged high performers

Since attention is an important component of WM performances, we used the Engagement Index (EI), an EEG-derived metric that reflects sustained attention and mental involvement (Kamzanova et al., 2014; Prinzel et al., 2003).

**FIGURE 5:**
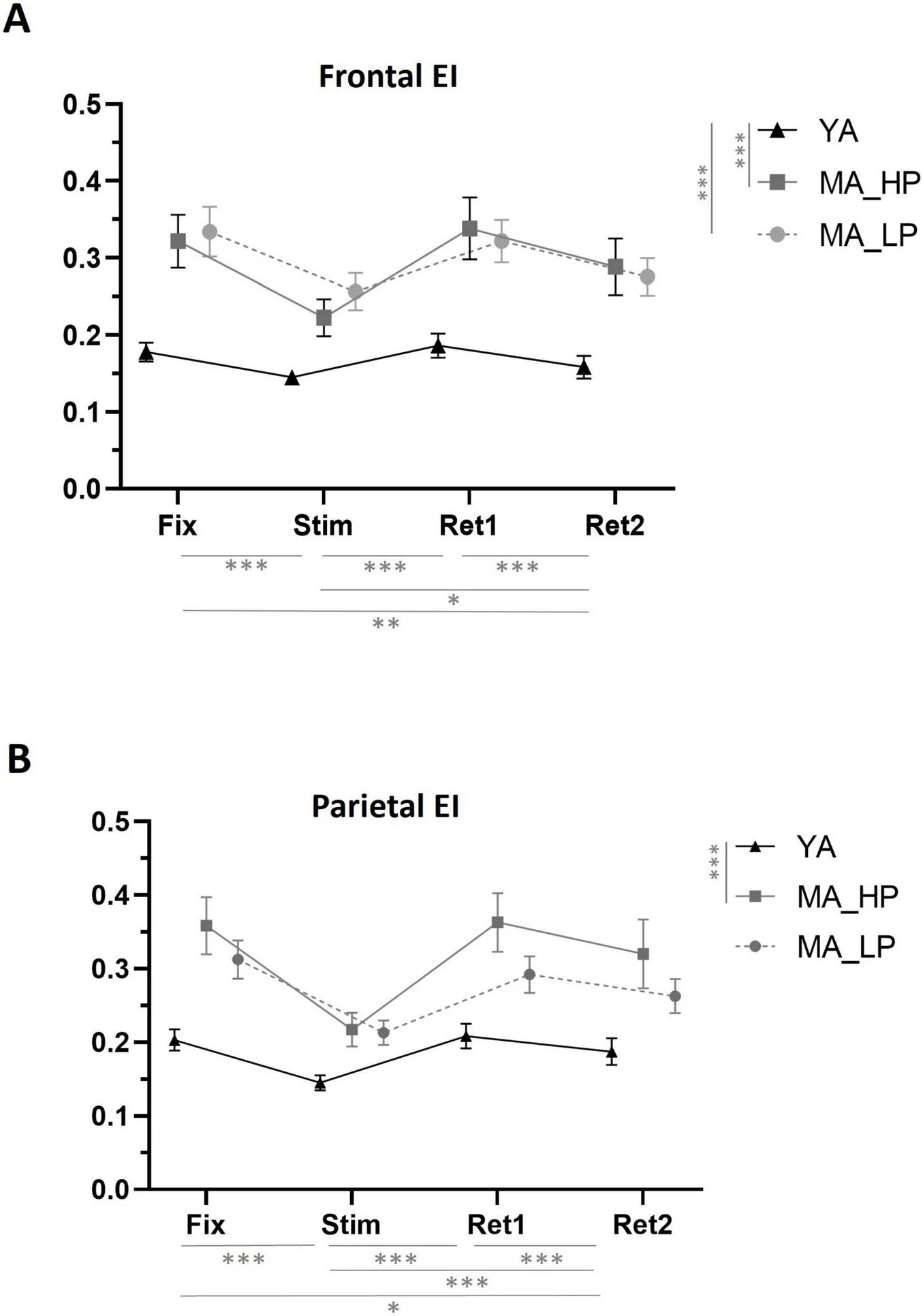
Indices of cognitive engagement. Engagement Index quantified during the four periods of the task in frontal **(A)** and parietal **(B)** areas. An age effect was observed at the frontal site whereas only the MA_HP displayed an increased parietal EI compared to YA during the retention period. Fix = fixation cross; Stim = stimuli presentation; Ret1 = first part of the retention and Ret2 = second part of the retention. Data are expressed as mean ± SEM. In absence of interaction between group effect and period effect, *post-hoc*-tests of single effects are represented close to the legends. *p < 0.05; **p < 0.001; ***p < 0.001. EI, Engagement Index; YA, young adults; MA_HP, Middle-aged high performers; MA_LP, Middle-aged low performers.

We analyzed EI using a mixed-design two-way ANOVA. Both frontal (Fz) and parietal (sum P1, Pz, P2) EI fluctuated during the trials [Figures 5A, B; main effect of trial period: F(2.26,179.17) = 28.853, *p* < 0.001; Greenhouse-Geisser corrected], and differed among groups [main group effect: F(2,65) = 8.533, *p* < 0.001]. No main effect of the brain area was observed suggesting that the EI was globally similar in the frontal and parietal areas [F (1,195) = 0.501, *p* = 0.482]. Interestingly, whereas the interaction trial period × group was not quite significant [F (4.52, 195) = 2.286, *p* = 0.055; Greenhouse-Geisser corrected], brain area × group interaction [F (2,195) = 5.769, *p* = 0.005] and brain area × trial period interaction [F (2.75,179.2) = 7.14, *p* < 0.001; Greenhouse-Geisser corrected] were both significant, indicating that engagement levels varied across brain regions depending on the participant group or trial phase. However, the absence of a significant three-way interaction (group × brain area × trial period; F(5.51,179.2) = 0.281, *p* = 0.936; Greenhouse-Geisser corrected) suggests that these effects did not jointly depend on all three factors.

*Post-hoc t*-tests indicated that both MA_HP and MA_LP groups exhibited higher EI values compared to YA group, highlighting an overall age-related increase in engagement. This effect was primarily driven by the frontal region, where both MA subgroups showed higher EI than YA (*p* ≤ 0.005). In contrast, in the parietal region, only MA_HP differed significantly from YA (*p* = 0.004), while MA_LP did not (*p* = 0.115).

EI also varied significantly across the different phases of the trial. It was high during the Fix period, decreased significantly during the Stim period, increased again during Ret-1, and dropped during Ret-2. This pattern of fluctuation (a high-low-high-low dynamic) was observed in both frontal and parietal regions.

Analysis of positive correlations between EI values and dilation values indicated a link between the frontal EI measured during Ret-2 and dilation values at each time point of the Ret-2 period (0.474 > R values > 0.385, *p* < 0.05) in HP_MA but not in LP_MA (data not shown).

Altogether, these results demonstrate that engagement shows dynamic variations throughout the trial, and is further modulated by age and performance, particularly in parietal brain regions.

### 3.4 Age and performance-related differences in event-related spectral perturbation (ERSP) during WM

To further investigate the neurophysiological underpinnings of WM performance in middle-aged participants, we analyzed Event-Related Spectral Perturbations (ERSP) in theta (4–8 Hz), alpha (8–12 Hz), and beta (13–25 Hz) bands across prefrontal (FPz), fronto-central (FCz), and parietal (Pz) electrodes during stimulus presentation and retention. ERSP captures task-related power changes relative to baseline, providing insights into oscillatory dynamics underlying WM processes. In the following text, “event related desynchronization” (ERD) was used when ERSP decrease reached significance compared to zero (one sample *t*-test, FDR corrected *p*-value for multiple comparisons; see Supplementary Table 1 for detailed *p*-values).

For all repeated-measures analyses of ERSP data described below, the assumption of sphericity was violated (Mauchly’s test, *p* < 0.05), thus all F and p values reported were Greenhouse-Geisser corrected. When the triple interaction (period × electrode × group) was present, we conducted planned follow-up analyses with a step down approach in order to disentangle this complex interaction (Field, 2012). Mixed-design ANOVAs (period × group) were conducted for each electrode to compare temporal trajectories between groups. Significance threshold was adjusted for multiple comparisons (3 electrodes) using the Bonferroni method (α = 0.05/3 = 0.0167). Detailed results of the statistical analyses are provided in Supplementary Tables 2–4 to avoid overloading the main text.

#### 3.4.1 Aging shifts theta desynchronization from parietal to prefrontal regions in successful midlife performers

Theta activity is closely linked to WM maintenance and executive control (Hsieh and Ranganath, 2014; Sauseng et al., 2010). In our study, theta ERD differed significantly between groups and scalp regions, as revealed by the three-way interaction in a mixed-design ANOVA [F (6.30, 198.32) = 2.48, *p* = 0.023].

**FIGURE 6:**
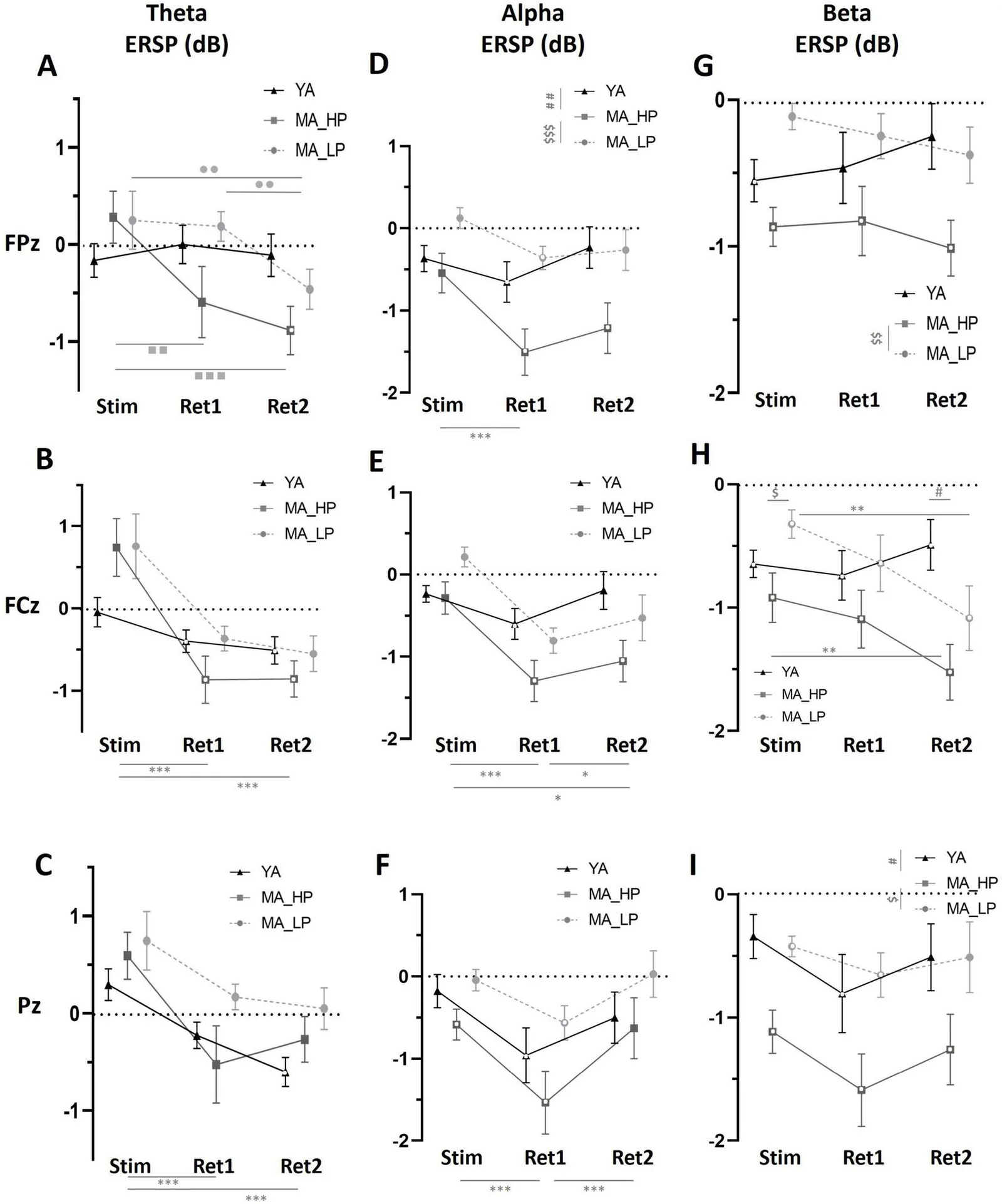
Event-related spectral perturbations during the visuo-spatial working memory task. Event-Related Spectral Perturbations (ERSP) quantified during stimuli presentation (Stim), first (Ret1) and second (Ret2) part of retention in the theta band (4–8 Hz) **(A–C)**, the alpha band (8–12 Hz) **(D–F)** and the beta band (13–25 Hz) **(G–I)** at FPz **(A, D**, **G)**, FCz **(B, E, H)** and Pz **(C, F, I).** Data are expressed as mean ± SEM. In absence of interaction between group effect and period effect, *post-hoc*-tests of single effects are represented close to the legends. Symbols with a white dot inside represent the values significantly different from baseline (fixation cross). The statistical values of the corresponding one-sample *t*-tests for comparison to the baseline are presented in Supplementary Table 1. *p* < 0.01 for between period comparison in MA_LP; *p* < 0.01; *p* < 0.001 for between period comparison in MA_HP. *p < 0.05; ***p < 0.001 for between period comparison independently of group; ^$^*p* < 0.05; ^$$^*p* < 0.01; ^$$$^*p* < 0.001; for MA_LP vs. MA_HP comparisons; ^#^*p* < 0.05; ^##^*p* < 0.01 for YA vs. MA_HP comparisons.

At FPz, theta ERSP showed time x group interaction [F (3.648, 114.927) = 4.14; *p* = 0.005]. Prefrontal theta activity remained stable throughout the task in YA, whereas both MA groups showed a progressive decrease during retention (Figure 6A). However, only MA_HP reached a significant ERD (*p* = 0.018), suggesting that prefrontal theta ERD specifically characterizes successful cognitive aging.

At FCz, theta ERSP showed a main effect of period [F (1.474, 92.849) = 29.119; *p* < 0.001]. In all groups, theta power decreased from stimulus onset to the end of retention (Figure 6B), but ERD was observed only in YA and MA_HP during retention (Supplementary Table 1), whereas MA_LP showed only a trend (*p* = 0.062).

Parietal theta power also revealed a main effect of period [F (1.796, 113.162) = 13.259; *p* < 0.001] (Figure 6C), with a decrease from stimulus to retention. However, only YA participants display ERD at the end of the retention period (*p* = 0.007).

In summary, prefrontal theta power modulation during WM showed distinct spatial and temporal trajectories depending on age and performance level, while in fronto-central and parietal areas, all groups exhibited a decrease in theta power. Yet YA and MA distinguished themselves by the presence (YA) or absence (MA) of parietal theta ERD during mental manipulation. Finally, while MA_LP never reach ERD whatever the areas considered, MA_HP showed instead prefrontal and fronto-central theta ERD.

#### 3.4.2 Alpha ERD throughout WM task is associated with high WM performance in middle-aged adults

Alpha activity showed a consistent task-related desynchronization across the three groups, especially during early retention (Figure 6D–F). Yet, the global analysis revealed a significant three-way interaction group × electrode × period [F (6.16, 193.97) = 3.53, *p* = 0.002)].

At FPz, alpha ERSP showed a main effect of group [F (2,63) = 9.364; *p* < 0.001] and of period [F (1.709, 107.669) = 6.055; *p* = 0.005] without interaction between the two factors. Prefrontal alpha power exhibited transient decrease and ERD at the beginning of retention period in all groups (Figure 6D), but only MA_HP showed sustained ERD until the end of retention (*p* = 0.002).

At FCz, alpha ERSP showed a main effect of period [F(1.554,97.914) = 15.221; *p* < 0.001] and group effect was almost significant [F(2,63) = 4.214; *p* = 0.019] without interaction between the two factors. Fronto-central alpha power followed a “V-shaped” trajectory, with a drop during early retention and partial recovery later in the trial (Figure 6E). Again, while all groups displayed ERD during the first part of retention, only MA_HP still showed ERD at the end of retention (*p* = 0.002). This indicates that frontal alpha suppression during WM retention is particularly robust in high-performing middle-aged adults.

Importantly, stronger frontal and fronto-central alpha ERD during early retention was associated with greater pupil dilation in middle-aged adults (−0.431 < R values < −0.269, *p* < 0.05), supporting a link between alpha suppression and cognitive effort.

At Pz, alpha ERSP showed a main effect of period [F (1.902,119.802) = 11.11; *p* < 0.001] Parietal alpha activity displayed a similar V-shaped fluctuation across all groups (Figure 6F), with a decrease during early retention and a rebound thereafter. All three groups exhibited alpha ERD during the first part of retention but only MA_HP showed alpha ERD during stimulus presentation.

In summary, alpha activity showed clear task-related desynchronization during the first part of retention across fronto-parietal areas in all three groups. MA_HP distinguish themselves from the two other groups by more sustained ERD across the periods of the trial, starting during stimuli presentation at the parietal site and lasting until the end of retention at prefrontal and fronto-central sites.

#### 3.4.3 Beta ERD distinguishes high- from low-performing middle-aged adults

Beta ERSP values remained negative, revealing sustained task-related desynchronization throughout the trial. Yet, the significant three-way interaction electrode × period × group [F (7.202, 226.865) = 2.679, *p* = 0.01], indicates that both magnitude and topography of beta ERSP differed across groups and trial phases (Figures 6G–I).

At FPz, beta power differed among groups [F (2,63) = 5.367; *p* = 0.007] but did not show period nor group x period interaction. Prefrontal beta ERSP decrease was significantly stronger in MA_HP, compared to MA_LP (*p* = 0.007). YA displayed ERD only during stimulus presentation, while MA_HP showed sustained ERD from stimulus onset to the end of retention. MA_LP never reached significant ERD (Figure 6G; Supplementary Table 1). This indicates that prefrontal beta ERD is a distinctive feature of high performance in midlife.

At FCz, beta ERD was present throughout trial periods in all groups (Supplementary Table 1). Yet, beta ERSP showed time x group interaction [F (3.899, 122.824) = 3.525; *p* = 0.010]. YA showed a stable ERSP across the trial, whereas in both middle-aged groups beta power decreased during retention (Figure 6H). This suggests that fronto-central beta power decrease becomes more pronounced during WM maintenance in middle-aged adults.

At Pz, beta power differed among groups [F (2,63) = 5.499; *p* = 0.006] but did not show period nor group x period interaction (Supplementary Table 3). Parietal beta activity decrease was strongest in MA_HP compared to the two other groups (Figure 6I). During stimulus encoding, only MA displayed ERD. During the first part of retention, all groups showed ERD, but only MA_HP maintained ERD until the end of retention period (Supplementary Table 1).

In summary, beta ERD discriminate MA_HP from MA_LP during a WM task. Sustained and robust beta ERD, especially in prefrontal and parietal regions, is linked to higher cognitive performance in middle-aged adults.

## 4 Discussion

The present study aimed to disentangle age-related and performance-related changes in neurophysiological markers during a VSWM task in midlife. We investigated different measures, notably using pupillometry and EEG which can inform on cognitive engagement, mainly attentional, to the task. Our data show that certain indices track chronological age, whereas others are related to the success or failure of WM abilities in midlife (summarized in Table 2).

**TABLE 2:** Summary table of the chronological aging profile and the successful cognitive aging profile.

Task period	Neurophysiological indices	YA profile	Chronological aging profile	Successful cognitive aging profile
Encoding (Stimuli presentation)	Engagement indice		• Increased frontal EI	
	Event related desynchronisation	• Frontal β ERD • Fronto-central β ERD	• Parietal β ERD	• Parietal α ERD
	Pupil dilation	• Early pupil dilation		• Young-like pupil dilation latency
Retention First part (0–1.2 s)	Engagement Indice		• Increased frontal EI	• Increased parietal EI
	Event related desynchronisation	• Fronto-central ⊖ ERD • General α ERD • Fronto-central β ERD • Parietal β ERD		• Pre-frontal β ERD
	Pupil dilation	• Large pupil dilation	• Reduced dilation peak	• Continuous dilation reaching young-like level
Retention Second part (1.2–3 s)	Engagement indice		• Increased frontal EI	• Increased parietal EI
	Event related desynchronisation	• Fronto-central ⊖ ERD • Parietal ⊖ ERD • Fronto-central β ERD	• Loss of parietal ⊖ ERD	• Pre-frontal ⊖, α, β ERD • Maintenance of fronto-central ⊖ ERD • Fronto-central α ERD • Parietal β ERD

The chronological aging profile corresponds to neurophysiological modifications found in all MA adults compared to YA, independently of their WM performance. The successful aging profile corresponds to modifications observed exclusively in MA_HP. YA, Young adults; MA_HP, Middle aged high-performer; MA_LP, Middle-aged low-performer.

### 4.1 Working memory at midlife

Average WM scores were lower in MA than in YA. For both age groups, accuracy was significantly lower in the free recall than in the recognition condition, consistent with the literature (Rhodes et al., 2019) and with higher cognitive demands of free recall. Recognition allows shallower encoding without loss of accuracy thanks to external cues, whereas free recall, lacking such cues and being more susceptible to interference, requires deeper encoding and active retrieval strategies (Craik and McDowd, 1987; Yonelinas, 2024, 2002). To note, the performance gap between YA and MA was more pronounced in free recall than in recognition condition. These results indicate a stronger age-related performance decline in more demanding conditions. Although such a decline under high cognitive load is well established in elderly populations, our findings show that it is already observable in individuals in their fifties, highlighting that age-related cognitive vulnerabilities may emerge earlier than previously recognized. Whereas YA performances were relatively homogeneous, the MA group displayed substantial interindividual variability, suggesting heterogeneous cognitive aging in midlife and underscoring the importance of characterizing neural markers of successful versus frailty cognitive aging at this early chronological aging stage.

### 4.2 Indicators of attentional engagement

#### 4.2.1 Pupil dilation

*Baseline pupil dilation*

A recent paper using concomitantly fMRI and pupillometry revealed that when pupil dilation before stimulus onset is larger, the cortical representation of the stimulus is more precise, and the authors concluded that spontaneous fluctuations of arousal impact the fidelity of information processing (Geurts et al., 2024). Furthermore, several studies reported that baseline dilation, corresponding to a preparatory attentional engagement, and its variability across trials was related to performance in WM tasks (Hong et al., 2014; Robison and Unsworth, 2019). In contrast with this literature, we did not observe any differences in baseline dilation or in baseline variability between YA, MA_HP and MA_LP. This might be explained by the high perceptual load of our VSWM task, since it has been shown in a “search for target” task that pupil baseline predicted attentional performance in low load but not in high load. This suggests that different attentional mechanisms are involved, one in which the LC-NE system plays a key role (low load) and one in which it is less relevant (high load) (Oliva, 2019).

To conclude, our observations indicate that all three groups (YA, MA_HP and MA_LP) presented similar attentional engagement into the task before stimulus presentation, and that baseline pupil dilation does not inform on the age or on performances in our VSWM task.

*Pupil dilation peak*

When considering the link between the dilation peak evoked by a cognitive task and the performances obtained, the literature provides inconsistent conclusions. Some studies on inter-individual differences have revealed that increased pupil dilation is associated with enhanced performance in updating tasks (Rondeel et al., 2015; van der Meer et al., 2010). In oddball and Simon tasks larger dilation was associated to faster reaction time (Zhunussova et al., 2025). The relationship between pupil dilation and performance in inhibition tasks seems more complex: while several studies suggested that pupil dilation relates to impaired inhibition performances (Laeng et al., 2011; Rondeel et al., 2015), a recent work showed that better Stroop interference scores correlated with pupil dilation in a high load WM task (Lenain et al., 2026).

In our 3 groups, the mean peak dilation showed only a main effect of age independent of WM accuracy, suggesting that the maximum dilation during the retention phase is already attenuated in the 50s. This result is in accordance with the literature reporting a reduced cognitive related dilation in elderly, even though this observation is usually made in older populations (El Haj et al., 2023; Van Gerven et al., 2004).

*Temporal dynamics of dilation*

Peak amplitude alone omits rich information contained in dilation time-courses observed along a trial period. YA displayed the canonical biphasic pattern of dilation with a prompt rise in pupil size beginning around 200 ms after stimuli onset -indexing resource mobilization- followed by a sustained plateau reflecting active maintenance (Kosachenko et al., 2023; Robison and Unsworth, 2019). In MA, the dynamic of pupil dilation differed according to the level of performance. MA_HP showed a similar trajectory as the one observed in YA, whereas MA_LP exhibited an increased latency to dilation. Although pupil dilation amplitude has frequently been associated with LC-NE system activity, the specific interpretation of pupil response onset latency remains less firmly established. Therefore, caution is required when linking this measure directly to LC-NE dysfunction. Nevertheless, previous studies suggest that age-related changes in pupil response timing may reflect alterations in autonomic and neuromodulatory regulation. For instance, Bitsios et al. (1996) reported age-related changes in light reflex latency and proposed that these effects could be related to altered sympathetic activity. More recently, Huang et al. (2024) showed that pupil response onset latency in a low cognitive demand paradigm remained relatively stable throughout adulthood before gradually increasing after around 60 years of age.

In this context, our findings suggest that delayed pupil response onset may be associated not only with chronological aging of the LC-NE system, but also with inter-individual differences in cognitive efficiency within the same age cohort. Alternative explanations should also be considered, including age-related differences in attentional allocation, processing speed, autonomic regulation, or broader neuromodulatory changes involving multiple neurotransmitter systems beyond the noradrenergic pathway alone.

In addition to the delayed pupil response, MA_LP also showed a lower plateau than YA and MA_HP. In a princeps study, Kahneman and Beatty (1966) demonstrated that dilation scales with memory load until resource limits are reached, after which it levels off or declines (Granholm et al., 1996; Unsworth and Robison, 2015). More recently, Robison and Unsworth (2019) showed that young adults with low WM performance had a lower pupil dilation plateau compared to those with higher performance. Our WM task could place MA_LP in front of an over-loading cognitive situation which could lead them to disengage the task facing excessive cognitive load, resulting in a lower plateau during retention (Zekveld and Kramer, 2014). However, when MA_LP were asked how many items (out of 4) they were able to maintain in memory, the number was not significantly different from what was reported by the MA_HP, what is not in favor of this hypothesis. Another explanation could be that despite the same memory span reported by both middle-aged groups, insufficient LC-NE engagement yields weak attention to each memorized item, producing both the reduced plateau and subsequent retrieval failure. Neuroimaging studies support this view. The LC structural integrity predicts higher attentional performance as well as biological brain maintenance in healthy older adults (Plini et al., 2021), and in aging it preferentially declines in its rostral portion (Liu et al., 2019). Moreover, it was recently reported that in high-performer elderly, increased rostral LC activity correlates with better fluid cognition (Riley et al., 2023). Finally, a recent animal study corroborates this causal link since noradrenergic neurons depletion in the LC of young mice induced impaired attention and WM abilities (Gargano et al., 2023).

#### 4.2.2 Fronto-parietal engagement

Another way to assess cognitive engagement dedicated to a task is to calculate EEG-based indices. Pupil dilation on the one hand, and EI on the other, provide complementary insights into cognitive engagement during a WM task. While pupil dilation reflects global autonomic arousal, EEG-based indices offer spatially resolved markers of cortical engagement, both being related to attentional resources allocation. Thus, combined use of EI with pupillometry strengthens the characterization of cognitive engagement in midlife.

Regarding the EI, defined here as β/(α + θ), it has been proposed to be related to cognitive processes such as information processing and attention resources (Kamzanova et al., 2014). In the present study, EI rose from encoding to retention, suggesting increasing engagement as maintenance demands grew. Notable differences have been observed between the frontal and parietal sites. Frontal EI was higher in MA than in YA, indicating an age effect, whereas parietal EI increased significantly only in MA_HP compared to YA. To our knowledge, no study has investigated EEG indices in the context of a WM task. However, our results match with findings from highly demanding real-world tasks such as complex piloting in which EI scales with performance (Coelli et al., 2018; Dehais et al., 2018).

Elevated frontal EI in midlife fits the posterior–anterior shift in aging (PASA) model proposing that with age, processing migrates anteriorly (Davis et al., 2008). Lower EI in young adults in both frontal and parietal sites suggests that they reach automatization of the processing, thus lowering the load on attentional resources. Crucially, MA_HP also boosted parietal EI, whereas MA_LP did not, implying that simultaneous frontal and parietal engagement is needed to preserve WM accuracy. This is coherent with the literature investigating brain activation during a WM task. Indeed, even though the precise sites of activation may depend on the material manipulated during the task, WM is related to the activation of a fronto-parietal network. The fronto-parietal network underpins both attention and WM (LaBar et al., 1999; Naghavi and Nyberg, 2005). Aging appears to reduce the network’s dynamic range: older adults up-regulate activity at low load (1-back) but fail to scale further at high load (3-back) (Heinzel et al., 2017). Our EI results extend these observations to healthy MA adults: those who cannot raise parietal EI in tandem with frontal EI already show WM deficits.

It is also worth comparing these EI results with those of pupillary dilation. While EI, derived from brain oscillatory activity, reflects cognitive work-load, pupil dilation informs more on attentional resources mobilization.

In MA_HP, the higher frontal and parietal EI may reflect increased cortical recruitment needed to maintain a high level of performance in the task compared to young-adults. The continuous dilation during the whole retention period indicates that this increased cortical recruitment is associated to a sustained attentional mobilization. In contrast, in MA_LP, increased parietal engagement is lacking and pupil dilation reaches a low plateau indicating limited attentional resources mobilization leading to poor performances.

Moreover, beyond the noradrenergic system commonly associated with pupil dilation, the EEG changes observed during aging may also reflect the involvement of other neuromodulatory systems. In particular, the cholinergic system, which plays a key role in attention and cortical oscillatory modulation (Platt and Riedel, 2011) may contribute to age-related alterations in alpha and theta activity during working memory. Overall, the EEG differences between young adults and middle-aged HP and LP participants likely reflect the combined influence of multiple neuromodulatory systems involved in cognitive control and mental effort regulation.

### 4.3 Brain power spectrum modulations in the WM network

We also captured event-related spectrum perturbation over time within trials (ERSP). Fronto-medial theta increases are typically associated with attention and WM load (Nakamura-Palacios et al., 2023), yet reductions in theta power under cognitive demand are less well understood. Some studies in older adults report a frontal theta decrease with increased task difficulty (Cummins and Finnigan, 2007; McEvoy et al., 2001), possibly reflecting an age-related difficulty in sustaining attentional control. Additionally, theta activity tends to become more spatially diffuse with age (Mitchell et al., 2008).

Our findings suggest that fronto-central theta ERD during retention may support high WM performance, as it was observed in both YA and MA_HP but not in MA_LP. The additional prefrontal ERD seen only in MA_HP could reflect a compensatory mechanism engaged to support executive control processes. This interpretation is consistent with Bastiaansen et al. (2002), who found a sustained frontal theta decrease specifically linked to visuo-spatial information retention in adults, distinguishing memory-related processing from purely sensory processing.

Alpha and beta ERD are well-established markers of active information processing (Hanslmayr et al., 2012), interpretated either as a release from inhibition (Inhibition Theory) (Jensen and Mazaheri, 2010; Klimesch et al., 2007) or as cortical activation from an “idling” state (Idling Theory) (Neuper and Pfurtscheller, 2001; Pfurtscheller et al., 1996).

In all groups, alpha and beta ERD were observed over fronto-parietal regions. However, MA_HP showed the strongest and most sustained ERD, emerging in parietal area during encoding and spreading frontally throughout retention. This pattern suggests that parietal ERD supports visuo-spatial encoding, possibly linked to imagery-based strategies (self-reported by participants), while frontal ERD during retention reflects executive monitoring of WM contents. The broad fronto-parietal beta ERD likely facilitates large-scale communication across regions, enabling flexible cognitive engagement.

These findings are consistent with the PASA model (Davis et al., 2008), whereby prefrontal recruitment compensates for declining posterior efficiency. Together, our data suggest that successful middle-aged individuals implement both improved encoding strategies and executive-controlled maintenance, enabling them to preserve “young-like” WM performance despite age-related neural changes.

Interestingly, ERSP data show that brain power spectrum modulations are not stable through the retention period. This is coherent with the biphasic pattern observed in pupil dilation suggesting different cognitive processes during the first and second part of the retention. The initial increase in pupil size may reflect cognitive effort associated with active manipulation and stabilization of the visuospatial representation immediately after stimulus encoding. In contrast, retention period 2 may correspond to a state of information maintenance. These differences in cognitive operations across the retention period are likely associated with distinct neural oscillatory mechanisms, which could explain the different EEG changes observed between the two retention periods. Compared to both YA and MA_LP, MA_HP were characterized by a global pre-frontal ERD specifically the second part of the retention period. This could be an important support for an efficient maintenance of the information during the retention allowing a correct recall.

### 4.4 A unified view of our results

Our findings converge to show that high WM performance in midlife relies on the dynamic interplay between autonomic engagement (reflected by pupil dilation) and cortical modulation within fronto-parietal areas (indexed by EEG).

Our physiological data suggest that executive control is important for successful aging: MA_HP exhibited increased prefrontal engagement during the trial, associated with parietal activation during encoding. These spatial and temporal modulations of prefrontal and parietal areas activity suggest the implementation of adaptive, compensatory mechanisms that sustain performance through modulation of attentional and or executive processes.

A central player in this fronto-parietal modulation may be the LC-NE system, indirectly “captured” here through pupil dynamics. Indeed, this brainstem nuclei sends noradrenergic projection to all cortical regions but it is also the only source of NE for the cortex (for review see Sara and Bouret, 2012). Pupil dilation patterns in MA_HP closely resembled those of YA, both in amplitude and timing, suggesting a preserved LC-NE responsiveness. Notably, we observed that during retention, pupil dilation positively correlated with both frontal and parietal EEG engagement (EI) in MA_HP – but not in MA_LP – pointing to a functional coupling between cortical and subcortical systems in high performers. This coupling was also evident in the relationship between pupil dilation and alpha ERD. In MA_HP, early parietal alpha ERD during encoding and sustained frontal alpha ERD during retention were both related to optimal pupil dynamics. In contrast, MA_LP showed delayed and reduced pupil responses, accompanied by insufficient alpha ERD – particularly in prefrontal regions – possibly explaining their performance deficits.

Taken together, these results support the view of a compensatory recruitment of cortical resources associated to the functional maintenance of subcortical systems, allowing MA_HP to exhibit “youth-like” WM performance. This emphasizes the need to consider inter-individual variability in cognitive aging studies, particularly in the understudied age range of the fifth decade – a critical period when early signs of divergent cognitive trajectories may first emerge. By focusing on this often-overlooked age group, we were able to identify two distinct patterns: (1) age-related changes attributable to chronological aging alone, and (2) a distinct, adaptive modulation within the fronto-parietal WM/attention network that is unique to middle-aged individuals who display WM performance at youth levels. This compensatory mechanism likely facilitates the mobilization of sufficient attentional support for an efficient WM processing (see Table 2 for an overview of these adaptive phenomena). These results offer a rare glimpse into the neurophysiological signatures that distinguish individuals with high versus low WM performance as early as their 50s.

## 5 Limitations

This study has several limitations. First, our sample size remains relatively modest, limiting the statistical power and generalization of our conclusions. Second, while pupil dilation is a well-established indirect marker of LC-NE activity, we acknowledge that direct measurements of LC-NE function (e.g., fMRI, PET) would be needed to confirm this link.

Another critical limitation of our cross-sectional design is that we cannot determine whether the differences between MA_HP and MA_LP groups reflect age-related cognitive changes or pre-existing individual differences in WM ability. While the greater variability in WM performance among middle-aged adults (coefficient of variation: 40.6% vs. 19.0% in YA) and the youth-like neurophysiological profiles of MA_HP (e.g., pupil dilation dynamics, parietal engagement) suggest that cognitive aging processes are at play, an alternative explanation is that MA_HP and MA_LP may have always differed in their neural and cognitive functioning. Only longitudinal studies tracking individuals from young adulthood to midlife could definitively distinguish between these interpretations. Until then, our findings should be interpreted with caution, acknowledging that both age-related and trait-like differences may contribute to the observed patterns.

We are currently conducting annual follow-ups of our middle-aged participants to confirm whether the neural profiles we highlighted are stable predictors of cognitive coping or precursors of larger decline.

Beyond this, further exploration of the brain’s adaptive potential could benefit from more advanced analyses. We propose that investigating interhemispheric coherence and cross-frequency coupling within the fronto-parietal network would offer deeper insight into the attentional mechanisms supporting preserved WM in aging.

## 6 Conclusion

This study provides new insight into the early neurophysiological underpinnings of cognitive aging, showing that as early as midlife, decades before the typical onset of age-related decline, individuals already exhibit marked differences in their ability to sustain WM performance. Our findings challenge the notion that high performance in aging solely results from neural efficiency. Instead, we show that it relies on the flexible and adaptive mobilization of both cortical (fronto-parietal network) and autonomic (LC-NE system) resources in response to cognitive demands. These compensatory mechanisms appear to be a key feature of cognitive coping.

A key innovation of this work lies in its focus on the fifth decade of life, a critical yet understudied period for identifying early markers of cognitive resilience or vulnerability. By revealing these mechanisms, we not only advance our understanding of successful cognitive aging but also pave the way for early, non-invasive detection of at-risk individuals. Specifically, our results support the use of pupillometry as a simple, accessible tool to detect subtle autonomic changes linked to cognitive decline – long before behavioral symptoms manifest.

## Data Availability

The raw data supporting the conclusions of this article will be made available by the authors, without undue reservation.
