# Supplementary material for: Modulation of fronto-parietal and autonomic activity underpins preserved working memory in middle-aged adults
Source: Front Aging Neurosci. 2026 Aug 26;18:1838829. doi: 10.3389/fnagi.2026.1838829 (PMC13553888; doi:10.3389/fnagi.2026.1838829)
Supplement: Supplementary file 1 [file Data_Sheet_1.pdf]

Supplementary Table 1: Comparisons of ERSP values to the baseline

Statistical results of the one-sample t-tests performed to compare each ERSP value to zero in order to highlight significant variation from the baseline (see Figure 6). p values were corrected with FDR correction for 27 comparisons. $*p<0.05$; $**p<0.01$; $***p<0.001$. Significant p values indicate a significant Event-Related Depression (ERD). YA = Young adults; MA_HP = Middle aged high-performer; MA_LP = Middle-aged low-performer.

**Theta**		Stim	Ret1	Ret2
	YA	0.436	0.993	0.702
FPz	MA_HP	0.395	0.203	0.018*
	MA_LP	0.484	0.335	0.086
	YA	0.846	0.041*	0.038*
FCz	MA_HP	0.106	0.038*	0.007**
	MA_LP	0.139	0.062	0.062
	YA	0.160	0.203	0.007**
Pz	MA_HP	0.062	0.318	0.362
	MA_LP	0.062	0.318	0.846

**Alpha**		Stim	Ret1	Ret2
	YA	0.060	0.039*	0.427
FPz	MA_HP	0.063	0.002**	0.002**
	MA_LP	0.405	0.041*	0.382
	YA	0.060	0.017*	0.441
FCz	MA_HP	0.216	0.002**	0.002**
	MA_LP	0.137	0.002**	0.118
	YA	0.440	0.030*	0.176
Pz	MA_HP	0.017	0.002**	0.156
	MA_LP	0.767	0.035*	0.912

**Beta**		Stim	Ret1	Ret2
	YA	0.002**	0.087	0.279
FPz	MA_HP	0.001***	0.004**	0.001***
	MA_LP	0.227	0.132	0.084
	YA	0.001***	0.004**	0.039*
FCz	MA_HP	0.001***	0.001***	0.001***
	MA_LP	0.016*	0.016*	0.001***
	YA	0.087	0.03*	0.088
Pz	MA_HP	0.001***	0.001***	0.001***
	MA_LP	0.001***	0.002**	0.098
