# Supplementary material for: Modulation of fronto-parietal and autonomic activity underpins preserved working memory in middle-aged adults
Source: Front Aging Neurosci. 2026 Aug 26;18:1838829. doi: 10.3389/fnagi.2026.1838829 (PMC13553888; doi:10.3389/fnagi.2026.1838829)
Supplement: Supplementary file 2 [file Data_Sheet_2.pdf]

# Supplementary Table 2: Detailed results of the repeated-measures ANOVA for theta-band ERSP

## 1- FPz theta

| Cases | Sphericity Correction | Sum of Squares | df | Mean Square | F | p |
|---|---|---|---|---|---|---|
| Time | None | 12.191[a] | 2.000[a] | 6.096[a] | 9.466[a] | < .001** |
| | Greenhouse-Geisser | 12.191 | 1.824 | 6.683 | 9.466 | < .001** |
| Time ✻ group | None | 10.665[a] | 4.000[a] | 2.666[a] | 4.140[a] | 0.003* |
| | Greenhouse-Geisser | 10.665 | 3.648 | 2.923 | 4.140 | 0.005* |
| Residuals | None | 81.141 | 126.000 | 0.644 | | |
| | Greenhouse-Geisser | 81.141 | 114.927 | 0.706 | | |
| group | | 5.710 | 2 | 2.855 | 1.043 | 0.358 |
| Residuals | | 172.381 | 63 | 2.736 | | |

*Note.*α-value adjusted for comparing a family of 3 estimates. *** p < .017; ** p < .003**

*Post Hoc Comparisons - group ✻ Time - Conditional on group*

| groupe | | | Mean Difference | SE | df | t | $p_{bonf}$ | $p_{holm}$ |
|---|---|---|---|---|---|---|---|---|
| YA | Stim | Ret1 | -0.165 | 0.278 | 63 | -0.594 | 1.000 | 1.000 |
| | | Ret2 | -0.054 | 0.267 | 63 | -0.203 | 1.000 | 1.000 |
| | Ret1 | Ret2 | 0.111 | 0.211 | 63 | 0.525 | 1.000 | 1.000 |
| MA_HP | Stim | Ret1 | 0.875 | 0.265 | 63 | 3.299 | 0.005** | 0.003** |
| | | Ret2 | 1.166 | 0.254 | 63 | 4.584 | < .001*** | < .001*** |
| | Ret1 | Ret2 | 0.291 | 0.202 | 63 | 1.443 | 0.462 | 0.154 |
| MA_LP | Stim | Ret1 | 0.064 | 0.254 | 63 | 0.252 | 1.000 | 0.802 |
| | | Ret2 | 0.710 | 0.243 | 63 | 2.916 | 0.015* | 0.010** |
| | Ret1 | Ret2 | 0.646 | 0.193 | 63 | 3.347 | 0.004** | 0.004** |

*** p < .05, ** p < .01, *** p < .001**

## 2- FCz theta

| Cases | Sphericity Correction | Sum of Squares | df | Mean Square | F | p |
|---|---|---|---|---|---|---|
| Time | None | 50.875[a] | 2.000[a] | 25.437[a] | 29.119[a] | < .001** |
| | Greenhouse-Geisser | 50.875 | 1.474 | 34.520 | 29.119 | < .001 |
| Time ✻ group | None | 10.530[a] | 4.000[a] | 2.632[a] | 3.013[a] | 0.021 |
| | Greenhouse-Geisser | 10.530 | 2.948 | 3.572 | 3.013 | 0.035 |
| Residuals | None | 110.071 | 126.000 | 0.874 | | |
| | Greenhouse-Geisser | 110.071 | 92.849 | 1.185 | | |
| group | | 3.314 | 2 | 1.657 | 0.649 | 0.526 |
| Residuals | | 160.822 | 63 | 2.553 | | |

*Note.* α-value adjusted for comparing a family of 3 estimates: *** p < .017; ** p < .003**

*Post Hoc Comparisons - Time*

| | | Mean Difference | SE | df | t | Cohen's d | $p_{bonf}$ | $p_{holm}$ |
|---|---|---|---|---|---|---|---|---|
| Stim | Ret1 | 1.027 | 0.170 | 63 | 6.039 | 0.858 | < .001*** | < .001*** |

*Post Hoc Comparisons - Time*

| | | Mean Difference | SE | df | t | Cohen's d | $p_{bonf}$ | $p_{holm}$ |
|---|---|---|---|---|---|---|---|---|
| | Ret2 | 1.123 | 0.198 | 63 | 5.680 | 0.938 | < .001*** | < .001*** |
| Ret1 | Ret2 | 0.096 | 0.109 | 63 | 0.879 | 0.080 | 1.000 | 0.383 |

***** p < .001**

*Note.* Results are averaged over the levels of group.

# 3- Pz theta

| Cases | Sphericity Correction | Sum of Squares | df | Mean Square | F | p |
|---|---|---|---|---|---|---|
| Time | None | 26.680[a] | 2.000[a] | 13.340[a] | 13.259[a] | < .001* |
| | Greenhouse-Geisser | 26.680 | 1.796 | 14.853 | 13.259 | < .001* |
| Time ✻ group | None | 3.179[a] | 4.000[a] | 0.795[a] | 0.790[a] | 0.534[a] |
| | Greenhouse-Geisser | 3.179 | 3.592 | 0.885 | 0.790 | 0.522 |
| Residuals | None | 126.771 | 126.000 | 1.006 | | |
| | Greenhouse-Geisser | 126.771 | 113.162 | 1.120 | | |
| group | | 9.262 | 2 | 4.631 | 2.695 | 0.075 |
| Residuals | | 108.244 | 63 | 1.718 | | |

*Note.* α-value adjusted for comparing a family of 3 estimates: *** p < .017; ** p < .003**

*Post Hoc Comparisons - Time*

| | | Mean Difference | SE | df | t | Cohen's d | $p_{bonf}$ | $p_{holm}$ |
|---|---|---|---|---|---|---|---|---|
| Stim | Ret1 | 0.738 | 0.161 | 63 | 4.597 | 0.662 | < .001*** | < .001*** |
| | Ret2 | 0.818 | 0.202 | 63 | 4.039 | 0.733 | < .001*** | < .001*** |
| Ret1 | Ret2 | 0.080 | 0.159 | 63 | 0.501 | 0.071 | 1.000 | 0.618 |

***** p < .001**

*Note.* Results are averaged over the levels of group.
