# Supplementary material for: Modulation of fronto-parietal and autonomic activity underpins preserved working memory in middle-aged adults
Source: Front Aging Neurosci. 2026 Aug 26;18:1838829. doi: 10.3389/fnagi.2026.1838829 (PMC13553888; doi:10.3389/fnagi.2026.1838829)
Supplement: Supplementary file 3 [file Data_Sheet_3.pdf]

Supplementary Table 3: Detailed results of the repeated-measures ANOVA for alpha-band ERSP

1- FPz alpha

Cases	Sphericity Correction	Sum of Squares	df	Mean Square	F	p
Time	None	10.948[a]	2.000[a]	5.474[a]	6.055[a]	0.003*
	Greenhouse-Geisser	10.948	1.709	6.406	6.055	0.005*
Time ✻ group	None	4.409[a]	4.000[a]	1.102[a]	1.219[a]	0.306[a]
	Greenhouse-Geisser	4.409	3.418	1.290	1.219	0.307
Residuals	None	113.908	126.000	0.904		
	Greenhouse-Geisser	113.908	107.669	1.058		
group		30.733	2	15.367	9.364	< .001**
Residuals		103.385	63	1.641		

Note. P-value adjusted for comparing a family of 3 estimates: * p < .017; ** p < .003

Post Hoc Comparisons - Time

		Mean Difference	SE	df	t	Cohen's d	p_{bonf}	p_{holm}
Stim	Ret1	0.577	0.144	63	4.010	0.538	< .001***	< .001***
	Ret2	0.309	0.197	63	1.566	0.288	0.367	0.164
Ret1	Ret2	-0.269	0.152	63	-1.768	-0.251	0.246	0.164

Post Hoc Comparisons - group

		Mean Difference	SE	df	t	Cohen's d	p_{bonf}	p_{holm}
YA	MA_HP	0.670	0.229	63	2.930	0.624	0.014*	0.009**
	MA_LP	-0.252	0.224	63	-1.125	-0.235	0.794	0.265
MA_HP	MA_LP	-0.922	0.218	63	-4.221	-0.859	< .001***	< .001***

* p < .05, ** p < .01, *** p < .001

Note. Results are averaged over the levels of: Time

2- FCz alpha

Cases	Sphericity Correction	Sum of Squares	df	Mean Square	F	p
Time	None	21.300[a]	2.000[a]	10.650[a]	15.221[a]	< .001**
	Greenhouse-Geisser	21.300	1.554	13.705	15.221	< .001**
Time ✻ group	None	5.016[a]	4.000[a]	1.254[a]	1.792[a]	0.134[a]
	Greenhouse-Geisser	5.016	3.108	1.614	1.792	0.152
Residuals	None	88.159	126.000	0.700		
	Greenhouse-Geisser	88.159	97.914	0.900		
group		11.874	2	5.937	4.214	0.019
Residuals		88.758	63	1.409		

Note. P-value adjusted for comparing a family of 3 estimates: * p < .017; ** p < .003

Post Hoc Comparisons - Time

		Mean Difference	SE	df	t	Cohen's d	p_{bonf}	p_{holm}
Stim	Ret1	0.799	0.123	63	6.485	0.826	< .001***	< .001***
	Ret2	0.490	0.181	63	2.707	0.506	0.026*	0.017*
Ret1	Ret2	-0.309	0.127	63	-2.440	-0.320	0.053	0.018*

* p < .05, *** p < .001

Note. Results are averaged over the levels of: groupe

3- Pz alpha

Cases	Sphericity Correction	Sum of Squares	df	Mean Square	F	p
Time	None	21.910	2.000	10.955	11.110	< .001**
	Greenhouse-Geisser	21.910	1.902	11.522	11.110	< .001**
Time ✻ group	None	2.087	4.000	0.522	0.529	0.714
	Greenhouse-Geisser	2.087	3.803	0.549	0.529	0.706
Residuals	None	124.245	126.000	0.986		
	Greenhouse-Geisser	124.245	119.802	1.037		
group		18.071	2	9.035	2.917	0.061
Residuals		195.151	63	3.098		

Note. P-value adjusted for comparing a family of 3 estimates: * p < .017; ** p < .003

Post Hoc Comparisons - Time

		Mean Difference	SE	df	t	Cohen's d	p_{bonf}	p_{holm}
Stim	Ret1	0.751	0.157	63	4.797	0.578	< .001***	< .001***
	Ret2	0.098	0.191	63	0.514	0.075	1.000	0.609
Ret1	Ret2	-0.654	0.171	63	-3.819	-0.503	< .001***	< .001***

*** p < .001

Note. Results are averaged over the levels of: group
