# Supplementary material for: Modulation of fronto-parietal and autonomic activity underpins preserved working memory in middle-aged adults
Source: Front Aging Neurosci. 2026 Aug 26;18:1838829. doi: 10.3389/fnagi.2026.1838829 (PMC13553888; doi:10.3389/fnagi.2026.1838829)
Supplement: Supplementary file 4 [file Data_Sheet_4.pdf]

## Supplementary Table 4: Detailed results of the repeated-measures ANOVA for beta-band ERSP

## 1- FPz beta

| Cases | Sphericity Correction | Sum of Squares | df | Mean Square | F | p |
|---|---|---|---|---|---|---|
| Time | None | 0.052[a] | 2.000[a] | 0.026[a] | 0.072[a] | 0.931 |
| | Greenhouse-Geisser | 0.052 | 1.704 | 0.031 | 0.072 | 0.905 |
| Time ✻ group | None | 2.111[a] | 4.000[a] | 0.528[a] | 1.447[a] | 0.222 |
| | Greenhouse-Geisser | 2.111 | 3.409 | 0.619 | 1.447 | 0.229 |
| Residuals | None | 45.959 | 126.000 | 0.365 | | |
| | Greenhouse-Geisser | 45.959 | 107.372 | 0.428 | | |
| group | | 15.598 | 2 | 7.799 | 5.367 | 0.007* |
| Residuals | | 91.554 | 63 | 1.453 | | |

*Note.* P-value adjusted for comparing a family of 3 estimates: * p < .017

***Post Hoc Comparisons - groupe***

| | | Mean Difference | SE | df | t | Cohen's d | $p_{bonf}$ | $p_{holm}$ |
|---|---|---|---|---|---|---|---|---|
| YA | MA_HP | 0.480 | 0.215 | 63 | 2.232 | 0.563 | 0.087 | 0.058 |
| | MA_LP | -0.176 | 0.211 | 63 | -0.833 | -0.206 | 1.000 | 0.408 |
| MA_HP | MA_LP | -0.656 | 0.205 | 63 | -3.191 | -0.769 | 0.007** | 0.007** |

** p < .01

*Note.* Results are averaged over the levels of: Time

## 2- FCz beta

| Cases | Sphericity Correction | Sum of Squares | df | Mean Square | F | p |
|---|---|---|---|---|---|---|
| Time | None | 5.362 | 2.000 | 2.681 | 6.562 | 0.002** |
| | Greenhouse-Geisser | 5.362 | 1.950 | 2.750 | 6.562 | 0.002** |
| Time ✻ group | None | 5.760 | 4.000 | 1.440 | 3.525 | 0.009* |
| | Greenhouse-Geisser | 5.760 | 3.899 | 1.477 | 3.525 | 0.010* |
| Residuals | None | 51.475 | 126.000 | 0.409 | | |
| | Greenhouse-Geisser | 51.475 | 122.824 | 0.419 | | |
| group | | 12.120 | 2 | 6.060 | 3.034 | 0.055 |
| Residuals | | 125.847 | 63 | 1.998 | | |

*Note.* P-value adjusted for comparing a family of 3 estimates: * p < .017; ** p < .003

***Post Hoc Comparisons - group ✻ Time - Conditional on group***

| group | | | Mean Difference | SE | df | t | Cohen's d | $p_{bonf}$ | $p_{holm}$ |
|---|---|---|---|---|---|---|---|---|---|
| ECJ | Stim | Ret1 | 0.093 | 0.198 | 63 | 0.470 | 0.096 | 1.000 | 0.959 |
| | | Ret2 | -0.154 | 0.217 | 63 | -0.711 | -0.159 | 1.000 | 0.959 |
| | Ret1 | Ret2 | -0.247 | 0.190 | 63 | -1.302 | -0.256 | 0.593 | 0.593 |
| H_ECV | Stim | Ret1 | 0.174 | 0.189 | 63 | 0.922 | 0.180 | 1.000 | 0.360 |
| | | Ret2 | 0.604 | 0.207 | 63 | 2.917 | 0.624 | 0.015* | 0.015* |

***Post Hoc Comparisons - group ✻ Time - Conditional on group***

| group | | | Mean Difference | SE | df | t | Cohen's d | $p_{bonf}$ | $p_{holm}$ |
|---|---|---|---|---|---|---|---|---|---|
| | Ret1 | Ret2 | 0.430 | 0.181 | 63 | 2.372 | 0.444 | 0.062 | 0.042* |
| L_ECV | Stim | Ret1 | 0.318 | 0.181 | 63 | 1.757 | 0.328 | 0.252 | 0.084 |
| | | Ret2 | 0.763 | 0.198 | 63 | 3.846 | 0.787 | < .001*** | < .001*** |
| | Ret1 | Ret2 | 0.445 | 0.174 | 63 | 2.564 | 0.459 | 0.038* | 0.025* |

* p < .05, *** p < .001

*Note.* P-value adjusted for comparing a family of 3 estimates.

***Post Hoc Comparisons - group ✻ Time - Conditional on Time***

| Time | | | Mean Difference | SE | df | t | Cohen's d | $p_{bonf}$ | $p_{holm}$ |
|---|---|---|---|---|---|---|---|---|---|
| Stim | ECJ | H_ECV | 0.274 | 0.215 | 63 | 1.275 | 0.283 | 0.621 | 0.261 |
| | | L_ECV | -0.323 | 0.211 | 63 | -1.532 | -0.333 | 0.391 | 0.261 |
| | H_ECV | L_ECV | -0.597 | 0.205 | 63 | -2.906 | -0.616 | 0.015* | 0.015* |
| Ret1 | ECJ | H_ECV | 0.355 | 0.324 | 63 | 1.095 | 0.367 | 0.834 | 0.556 |
| | | L_ECV | -0.098 | 0.318 | 63 | -0.309 | -0.102 | 1.000 | 0.758 |
| | H_ECV | L_ECV | -0.453 | 0.310 | 63 | -1.463 | -0.468 | 0.445 | 0.445 |
| Ret2 | ECJ | H_ECV | 1.033 | 0.342 | 63 | 3.016 | 1.066 | 0.011* | 0.011* |
| | | L_ECV | 0.594 | 0.335 | 63 | 1.771 | 0.613 | 0.244 | 0.163 |
| | H_ECV | L_ECV | -0.438 | 0.327 | 63 | -1.340 | -0.453 | 0.555 | 0.185 |

* p < .05

# 3- Pz beta

| Cases | Sphericity Correction | Sum of Squares | df | Mean Square | F | p |
|---|---|---|---|---|---|---|
| Time | None | 5.141 | 2.000 | 2.571 | 3.880 | 0.023 |
| | Greenhouse-Geisser | 5.141 | 1.858 | 2.766 | 3.880 | 0.026 |
| Time ✻ group | None | 0.459 | 4.000 | 0.115 | 0.173 | 0.952 |
| | Greenhouse-Geisser | 0.459 | 3.717 | 0.123 | 0.173 | 0.943 |
| Residuals | None | 83.471 | 126.000 | 0.662 | | |
| | Greenhouse-Geisser | 83.471 | 117.079 | 0.713 | | |
| groupe | | 26.879 | 2 | 13.440 | 5.499 | 0.006* |
| Residuals | | 153.971 | 63 | 2.444 | | |

*Note.* P-value adjusted for comparing a family of 3 estimates: * p < .017

***Post Hoc Comparisons - group***

| | | Mean Difference | SE | df | t | Cohen's d | $p_{bonf}$ | $p_{holm}$ |
|---|---|---|---|---|---|---|---|---|
| ECJ | H_ECV | 0.769 | 0.279 | 63 | 2.757 | 0.686 | 0.023* | 0.015* |
| | L_ECV | -0.023 | 0.273 | 63 | -0.084 | -0.021 | 1.000 | 0.933 |
| H_ECV | L_ECV | -0.792 | 0.266 | 63 | -2.972 | -0.706 | 0.013* | 0.013* |

* p < .05

.

*Note.* Results are averaged over the levels of: Time
